# Insulin-Like Growth Factor-1 Supplementation Promotes Brain Maturation in Preterm Pigs

**DOI:** 10.1523/ENEURO.0430-22.2023

**Published:** 2023-04-13

**Authors:** Line I. Christiansen, Bo Holmqvist, Xiaoyu Pan, Kristine Holgersen, Sandy E. H. Lindholm, Nicole L. Henriksen, Douglas G. Burrin, David Ley, Thomas Thymann, Per Torp Sangild, Stanislava Pankratova

**Affiliations:** 1 Comparative Pediatrics and Nutrition, Department of Veterinary and Animal Sciences, Faculty of Health and Medical Sciences, University of Copenhagen, 1870 Frederiksberg, Denmark; 2 ImaGene-iT AB, Medicon Village, 223 81 Lund, Sweden; 3 US Department of Agriculture/Agricultural Research Service, and Department of Pediatrics, Baylor College of Medicine/Texas Children's Hospital, Children’s Nutrition Research Center, Houston, Texas 77030; 4 Department of Clinical Sciences Lund, Pediatrics, Lund University, 221 84 Lund, Sweden; 5 Department of Neonatology, Rigshospitalet, 2100 Copenhagen, Denmark; 6 Department of Pediatrics, Odense University Hospital, 5000 Odense, Denmark; 7 Department of Neuroscience, Faculty of Health and Medical Sciences, University of Copenhagen, 2200 Copenhagen, Denmark

**Keywords:** IGF-1, IGF1R, developing brain, hippocampus, cortex

## Abstract

Very preterm infants show low levels of insulin-like growth factor-1 (IGF-1), which is associated with postnatal growth restriction and poor neurologic outcomes. It remains unknown whether supplemental IGF-1 may stimulate neurodevelopment in preterm neonates. Using cesarean-delivered preterm pigs as a model of preterm infants, we investigated the effects of supplemental IGF-1 on motor function and on regional and cellular brain development. Pigs were treated with 2.25 mg/kg/d recombinant human IGF-1/IGF binding protein-3 complex from birth until day 5 or 9 before the collection of brain samples for quantitative immunohistochemistry (IHC), RNA sequencing, and quantitative PCR analyses. Brain protein synthesis was measured using *in vivo* labeling with [2H5] phenylalanine. We showed that the IGF-1 receptor was widely distributed in the brain and largely coexisted with immature neurons. Region-specific quantification of IHC labeling showed that IGF-1 treatment promoted neuronal differentiation, increased subcortical myelination, and attenuated synaptogenesis in a region-dependent and time-dependent manner. The expression levels of genes involved in neuronal and oligodendrocyte maturation, and angiogenic and transport functions were altered, reflecting enhanced brain maturation in response to IGF-1 treatment. Cerebellar protein synthesis was increased by 19% at day 5 and 14% at day 9 after IGF-1 treatment. Treatment had no effect on Iba1^+^ microglia or regional brain weights and did not affect motor development or the expression of genes related to IGF-1 signaling. In conclusion, the data show that supplemental IGF-1 promotes brain maturation in newborn preterm pigs. The results provide further support for IGF-1 supplementation therapy in the early postnatal period in preterm infants.

## Significance Statement

Deficiency of systemic insulin-like growth factor-1 (IGF-1) is associated with delayed neurologic development in preterm infants. Here, we show that the IGF-1 receptor is primarily expressed in immature neurons in the developing brain of the translational preterm pig model. Supplementation with IGF-1 accelerates neuron differentiation in the hippocampus and promotes myelination in subcortical white matter regions in a time-dependent way. Furthermore, systemic IGF-1 supplementation stimulates cerebral protein synthesis. Our study suggests that IGF-1 therapy in the early postnatal period might be supportive for neurodevelopment in preterm infants.

## Introduction

Insulin-like growth factor-1 (IGF-1) and its receptors are centrally involved in the regulation of fetal growth and organ development (Hansen-Pupp et al., 2007; Hellström et al., 2016). The level of IGF-1 in the serum of human fetuses is high during the last trimester and then decreases immediately after birth, followed by a gradual increase during the first postnatal weeks (Lineham et al., 1986; Langford et al., 1998; Bunn et al., 2005). Compared with full-term infants, neonates born very preterm (<32 weeks of gestation) have a sustained period of low-plasma IGF-1 levels (Lineham et al., 1986; Hellström et al., 2016), and a decrease in levels correlates with several morbidities, such as postnatal growth restriction (Kajantie et al., 2002), retinopathy of prematurity (ROP; Liegl et al., 2016), bronchopulmonary dysplasia (Hellström et al., 2003; Löfqvist et al., 2012), and necrotizing enterocolitis (NEC; Hansen-Pupp et al., 2011, 2013; Beardsall et al., 2014; Hellström et al., 2016). Additionally, low postnatal IGF-1 levels have been linked to reduced volumes of whole-brain, cerebellum, gray matter, and unmyelinated white matter (WM), together with reduced head circumference and impaired neurodevelopment (Hellström et al., 2016). However, cause–effect relationships between plasma IGF-1 and brain development in preterm infants remain unclear.

Circulating IGF-1 is mainly produced and secreted from the liver, and in fetuses and infants, its production is regulated by insulin (Hellström et al., 2016). Circulating IGF-1 is able to cross the blood–brain barrier, where neuronal activity stimulates brain region-dependent IGF-1 uptake (Carro et al., 2005; Nishijima et al., 2010). IGF-1 mediates signaling through high-affinity binding to IGF-1 receptor (IGF1R)/insulin receptor (InR) heterodimers. IGF1R is known to be widely expressed throughout the brain in neuronal progenitor cells, mature neurons, oligodendrocytes, and endothelial and neuroepithelial cells of the choroid plexus in both human and rodent brains (Marks et al., 1991; Garcia-Segura et al., 1997; Zeger et al., 2007; Suh et al., 2013; Ziegler et al., 2015; Labandeira-Garcia et al., 2017), but its expression in larger animals is unknown. The bioavailability of circulating IGF-1 and its half-life are modulated by at least six high-affinity IGF1-binding proteins (IGFBPs1–6; Clemmons, 2016), and the level of the most important of these, IGFBP3, follows the levels of plasma IGF-1 *in utero* (Langford et al., 1998). In addition to systemically derived IGF-1, this anabolic and neuroactive peptide is produced locally in the brain and is involved in a wide range of homeostatic and developmental processes including gliogenesis, oligodendrocyte maturation, myelination, axon growth, and regulation of cell survival (Bartlett et al., 1992; Beck et al., 1995; Chrysis et al., 2001; Zeger et al., 2007; Liu et al., 2009; Jin et al., 2019). Accordingly, the overexpression of IGF-1 in rodent brains resulted in brain enlargement, while the deletion of IGF-1 or conditional deletion of IGF1R expression in the brain led to microcephaly and hypomyelination (Beck et al., 1995; Zeger et al., 2007; Kappeler et al., 2008).

Our previous studies showed that preterm pigs have low IGF-1 levels in plasma, thus resembling preterm neonates. Furthermore, the treatment of preterm pigs with exogenous IGF-1 tended to reduce the incidence and severity of NEC (Andersen et al., 2016; Holgersen et al., 2020).

Given the high risk of long-term major neurodevelopmental disorders associated with prematurity, including cerebral palsy and intellectual disability (Soleimani et al., 2014), there is a distinct need to identify novel therapeutic strategies to improve brain development in preterm infants. Here, we hypothesized that exogenous IGF-1 can improve brain maturation. To test our hypothesis, we supplemented preterm pigs with IGF-1/IGFBP-3 at normal physiological levels and analyzed the developing brain at day 5, after an intense period of physiological adaptation to life *ex utero*, and then again at day 9, when preterm pigs showed more advanced motor development (Sangild et al., 2013; Andersen et al., 2016; Bæk et al., 2021).

## Materials and Methods

### Animal model

All animal procedures were performed in accordance with the Danish National Committee on Animal Experimentation (license no. 2014–15-0201–00418) and complied with ARRIVE (Animal Research: Reporting of In Vivo Experiments) guidelines.

Preterm piglets (Danish Landrace × Yorkshire × Duroc) were delivered on postnatal day 1 (P1) from five sows by cesarean section at day 106 of gestation (term = 117 d). Following resuscitation, they were housed in individual oxygenated and temperature-regulated incubators and reared to either P5 or P9. Pigs were stratified by birth weight and sex and were randomly allocated to two groups treated with either recombinant human (rh)IGF-1/rhIGFBP-3 complex (2.25 mg/kg/d; mecasermin rinfabate, Takeda), hereafter indicated as IGF-1, or vehicle (control group), hereafter indicated as CON. The treatments were delivered either as continuous systemic infusion via an intra-arterial cord catheter until P5 or subcutaneously three times daily until P9. Selected doses and administration routes were based on a previous pharmacokinetic study in preterm pigs shown to elevate plasma IGF-1 levels within physiological levels for young pigs (Holgersen et al., 2020). The used modes of administration (systemic and/or subcutaneous) are both relevant for routine administration to preterm infants, if/when ongoing clinical trials are successful (ClinicalTrials.gov Identifiers NCT03253263 and NCT01096784, using systemic infusion). Within 1–3 h after delivery, pigs were fitted with an orogastric feeding tube and an arterial umbilical catheter for enteral and parenteral nutrition, respectively, and fed every 3 h with gradually increasing volumes of infant formula (16–80 ml/kg/d for 4 d or 16–96 ml/kg/d for 8 d) supplemented by continuous parenteral nutrition via the cord catheter at a constant rate of 120 ml/kg/d for 4 d or 96–144/ml/kg/d for 8 d, using a commercially available parenteral nutrition product (Kabiven, Fresenius Kabi) modified as described previously (Sangild et al., 2013). The composition of the formula was 25 g/L Fantomalt, 2 g/L Phlexy-Vits, 25 g/L MIPRODAN 40, 55 g/L Lacprodan DI-9224, 43 g/L Liquigen MCT, and 30 g/L Calogen LCT. Detailed physiological and clinical responses to IGF-1 treatment, including gut, metabolic, and immune effects, are reported in a separate article (Holgersen et al., 2022).

### Behavior analysis

Neuromotor scoring and open field testing were performed on P7 using an open-field square arena (120 × 120 cm) surrounded by black wooden walls and placed in a secluded room bordered by curtains to shield from any outside disturbances. Video recordings from the arena were subdivided into a central square, peripheral areas, and corners [left bottom (LB), right bottom (RB), right top (RT), left top (LT)] to assess zone-dependent activities, as illustrated in Extended Data Table 1-1*A*. To assess general locomotor activity level and willingness to explore the environment, animals were individually placed in the middle of the arena and allowed to walk freely for 4 min while being recorded by a bird’s-eye view-placed camera. The arena was cleaned with soap water between animals. The total distance traveled, and the velocity and time spent in different areas of the arena were analyzed with the video-tracking software (EthoVision XT10, Noldus Information Technology).

For neuromotor scoring, two independent observers scored the pigs for the ability to stand, motor activity (walk vs stand/sit), gait and posture (normal vs asymmetric/stiff), head position (normal vs tilt), and anxiety (relaxed vs fearful/tense/stressed) every 30 s during the first 2 min of the open field arena exploration, with each parameter scored as absent = 0 or clearly present = 1. Scores for each pig were summarized. Animals incapable of getting on their feet at P7 were omitted from the open field test and neuromotor assessments. All behavioral tests and analyses were performed by researchers blinded to the treatment groups.

### Tissue collection

On P5 or P9, the pigs were anesthetized and then killed with intracardiac injection of sodium pentobarbital. The brain was weighed and hippocampal tissue was isolated from the left hemisphere and snap frozen in liquid nitrogen and stored at −80°C. The entire right brain hemisphere was immersed in 4% paraformaldehyde (PFA) made in PBS, 0.1 m, pH 7.4, for 48 h, divided transversally into four tissue slabs, according to a pig brain stereotaxic atlas (Félix et al., 1999), and fixed for an additional 6 h in PFA. The slabs were dehydrated and embedded in paraffin for coronal sectioning. The brain water fraction was estimated by weighing the remaining left hemisphere before and after drying the tissue until the weight was constant in an oven at 45°C. In addition to 11–12 brains per group at P5 and P9, brains from seven preterm newborn pigs were collected at P1 for immunohistochemical analysis. Gut pathology (NEC) was scored macroscopically in the colon and the proximal, middle, and distal small intestine by two independent investigators immediately after the pigs were killed, as described previously (Holgersen et al., 2020).

CSF was collected by suboccipital puncture immediately after the pigs were killed, centrifuged at 2500 × *g* and 4°C for 10 min, and stored at −80°C. The concentration of IGF-1 in plasma samples collected at both P5 and P9 and in CSF samples collected at P5 was measured by an IGF-1 ELISA kit (E20, Mediagnost).

### Brain protein synthesis *in vivo*

Fractional rates of protein synthesis were measured with a flooding dose (10 ml/kg) of l-phenylalanine (Phe; 1.5 mmol/kg) containing l-[ring-^2^H_5_]Phe at 30 mol % (0.45 mmol/kg; Cambridge Isotope Laboratories) injected intra-arterially 30 min precisely before the pigs were killed. Collected cerebellum samples were snap frozen in liquid nitrogen and stored at −80°C. Analysis for tracer enrichment in the brain and calculation of fractional rates of protein synthesis (Ks; percentage of protein mass synthesized/d) were performed as described previously (Rudar et al., 2021).

### Immunohistochemistry and imaging data processing

Paraffin-embedded brain tissue slabs containing the hippocampus were serially sectioned (5 μm) on a rotating microtome (Microm HM 360, Microm International). Sections collected on microscope slides (Superfrost Plus, Menzel-Glaser) were deparaffinized and rehydrated in graded ethanol followed by washes in PBS. Cresyl violet staining was performed for neuroanatomical evaluation of collected sections to ensure the presence of corresponding regions of interest (ROIs). For immunohistochemistry (IHC) labeling, heat-mediated epitope retrieval was performed in 0.01 m sodium citrate buffer, pH 6, containing 0.05% Tween-20, which was heated in a microwave oven to 95°C for 30 min. After quenching endogenous peroxidase in 0.3% hydrogen peroxide (H_2_O_2_; MilliporeSigma Merck) for 10 min, sections were blocked with 1% BSA dissolved in PBS containing 0.05% Triton X-100 (PBSTX-BSA) and were then incubated for 16 h at 4°C with primary antibody (Extended Data Fig. 1-1) diluted in PBSTX-BSA, while for doublecortin (DCX) labeling, the primary antibody incubation time was 70 min. The PBS-rinsed sections were incubated with species-specific HRP-conjugated secondary antibodies (Extended Data Fig. 1-1) for 30 min at room temperature (RT), rinsed in PBSTX, and then incubated in a diaminobenzidine (DAB; Sigma-Aldrich) solution containing 0.1% H_2_O_2_ for 10 min at RT. Sections were nuclear counterstained with Mayer’s hematoxylin (HTX; Histolab), dehydrated in rising alcohol series ended with xylene (100%) and coverslipped with Pertex Mounting Medium (Histolab). As antibody-binding controls, adjacent sections were processed simultaneously but excluded the primary antibody incubation from the labeling protocol. A minimum of two sections per animal from two different levels were analyzed. Sections from 11–12 animals per treatment group at each time point (i.e., P5 and P9) were labeled and analyzed.

Digital images were obtained at 40× magnification using a slide scanner (model NanozoomerS60, Hamamatsu Photonics). To analyze selected brain structures, the specific ROI was delineated on digital images using a free-form polygon annotation tool (NDP.view 2 software, Hamamatsu Photonics). The anatomic location of each ROI was identified based on a pig brain atlas (Félix et al., 1999; Saikali et al., 2010). The ROIs were extracted from the digital images and analyzed with ImageJ 1.53c using the Fiji extension. Quantification of the fraction of the immunoreactive area (area percentage) displaying an intensity value above background levels was performed for each traced ROI for synaptophysin (Syn), myelin basic protein (MBP), DCX, allograft inflammatory factor 1 (Iba1), oligodendrocyte transcription factor 2 (Olig2), and neuronal-specific nuclear protein (NeuN) labeling using the color deconvolution function for DAB-brown and HTX-blue, and normalized to the total area of the ROI. The number of labeled cells (cell percentage) in each ROI was further quantified for Olig2 and NeuN staining using Fiji default autothreshold segmentation algorithm, watershed separation, and smoothing of boundary, and was normalized to the area of the ROI.

To acquire density color-coded heat maps of IGF1R labeling, a color deconvolution was performed in ImageJ using H and DAB settings. The resulting brown image (DAB) containing 255 different intensity values was digitally divided into four levels (red, green, blue, and black) each covering 25% of total intensity, where the red color represented the strongest DAB pixel intensity (green, medium; blue, low; black, background).

For immunofluorescence double labeling, sections were pretreated as described above and incubated overnight at 4°C using a mixture of two primary antibodies made in different species followed by incubation with a mixture of two fluorophore-conjugated species-specific secondary antibodies (Extended Data Fig. 1-1). Sections were counterstained with DAPI (Thermo Fisher Scientific) and coverslipped in antifade solution. Confocal laser-scanning microscope analyses were performed (Model LSM800 Microscope, Zeiss) using a 20× dry lens or a 40× or 63× oil-immersion lenses. Detection levels were set for each channel in the adjacent control sections lacking primary antibody incubation.

### RNA sequencing

Considering that NEC may affect hippocampal gene expression in preterm pigs (Sun et al., 2018), we only assessed gene expression in pigs not having any gut lesions (*n* = 10 randomly selected for each group at P5). The frozen hippocampus was freeze fractured by a cryogenic tissue pulverizer and homogenized in QIAzol Lysis Reagent (QIAGEN). Total RNA was isolated using the RNeasy Lipid Tissue Mini Kit (QIAGEN), and constructed libraries were sequenced on the Illumina NovaSeq platform with paired-end 150 bp read production (Novogene Europe). Quality and adapter trimming of raw reads was performed using TrimGalore (Babraham Bioinformatics). The remaining clean reads were aligned to the porcine genome (Sscrofa11.1) using HISAT2 (Kim et al., 2015). The annotated gene information of the porcine genome was downloaded from Ensembl (release 99), and the script *htseq-count* (Anders et al., 2015) was used to generate a gene count matrix.

### Gene expression by real-time quantitative PCR

RNA was isolated, as described for the RNA-sequencing (RNA-seq) method, from hippocampi collected on P5 and P9 from animals without lesions in the gut. Total RNA was reverse transcribed to cDNA with a high-capacity cDNA reverse transcription kit (Thermo Fisher Scientific) according to the manufacturer instructions. Gene expression analysis was performed by quantitative real-time PCR analysis using a LightCycler 480 SYBR Green I Master kit on a LightCycler 480 PCR platform (both from Roche). Porcine-specific primers, listed in Extended Data Figure 6-1, were designed with Primer-BLAST software (National Center for Biotechnology Information). Relative expression of target genes was determined and normalized to the housekeeping gene hypoxanthine-guanine phosphoribosyltransferase 1 (*HPRT1*) using the 2^-△△CT^ method.

### Statistics

Using the R software package (version 4.0.3; R Foundation), the effect of IGF-1 supplementation on IGF-1 plasma level, protein synthesis rate, hippocampal gene expression, and behavioral analyses were analyzed for each specific time point separately (i.e., at P5, P9 and behavior at P7), using multiple linear regression (lm function) with litter, birth weight, and sex as covariates to correct for any confounding effects of these variables. For IHC analysis, gut pathology was additionally included as an interaction term because of previous studies showing association between gut pathology and these outcomes (Sun et al., 2018). The effects of covariates and interaction term with gut were reported when significant. The effect of age on cortical and hippocampal levels of the Syn immunoreactive area was analyzed separately for each treatment group using multiple linear regression with the above-mentioned covariates and interaction term. Model validation was performed by testing the normality and homoscedasticity of the residual and fitted values. Data were transformed using log transformation when required, and a nonparametric analysis was performed when data did not fit the model. Correlation analysis was performed with Pearson R correlation using GraphPad Prism (version 7.0.0; GraphPad Software). Data are visualized with GraphPad Prism or R software and are presented as the mean ± SEM except quantitative PCR (qPCR) data, which is presented as the relative expression compared with controls. *p *<* *0.05 was considered statistically significant, and 0.05 < *p *≤* *0.1 was reported as a tendency toward an effect. For RNA-seq data, significant differentially expressed genes (DEGs) were identified by DESeq2 (Love et al., 2014) using a threshold of Benjamini–Hochberg test-adjusted *p* value < 0.1.

## Results

### Short-term treatment with IGF-1 does not affect motor development or brain growth

Pigs treated with IGF-1 showed significantly higher levels of IGF-1 in plasma samples collected at P5 and P9 (70.9 ± 4.1 and 91.0 ± 11.8 ng/ml, respectively) than the corresponding control pigs (27.4 ± 4.2 and 19.9 ± 2.6 ng/ml; both *p *<* *0.001; Holgersen et al., 2022). Levels of IGF-1 in the CSF were below the detection limit of the assay for both treated and control animals.

In contrast to term newborn pigs, which can walk within minutes of birth, 90% gestation preterm pigs show delayed ability to stand, and walking does not start before P3 to P6 (Holme Nielsen et al., 2018). The open field test was therefore performed on P7, testing only the pigs that were able to stand at this time (Extended Data Table 1-1*B*). Mean velocity, total traveled distance, time moving, and percentage of time spent in different areas of the arena were not different between the CON and IGF-1 groups (Table 1, Extended Data Table 1-1*B*,*C*). Neuromotor development assessed by a cumulative neuromotor score (for details, see Materials and Methods) also showed no differences between groups, suggesting no effect of IGF-1 supplementation on the early development of motor activity (at least in the short term). Birth weight tended to positively affect the neuromotor score (*r *=* *0.43, *p *=* *0.055; Extended Data Table 1-1*D*). Comparisons of absolute or relative total brain weights and weights of different brain regions showed no differences between treatments at either P5 or P9 (Table 2), indicating no effect of the treatment on body and gross brain growth parameters. The brain water fraction did not differ between treatment groups but was reduced in female pigs compared with male pigs at P9 (*p *=* *0.03).

**Table 1:** Variables recorded in the open field test with preterm pigs at postnatal day 7

Parameter	Unit	Control (*n* = 9)	IGF-1 (*n* = 12)	*p*
Mean velocity	cm/s	5.92 ± 1.33	6.38 ± 1.02	0.73
Distance traveled	cm	708.28 ± 159.34	762.13 ± 121.27	0.74
Time moving	s	78.59 ± 9.70	79.17 ± 6.99	0.89
Time not moving	s	41.16 ± 9.72	40.47 ± 7.09	0.85
Time in center	%	45.16 ± 9.74	27.47 ± 9.47	0.48
Time in border area	%	54.84 ± 9.74	72.53 ± 9.47	0.48
Time in zone LB	%	1.09 ± 1.09	8.98 ± 3.06	0.10
Time in zone LT	%	9.27 ± 4.77	13.50 ± 4.11	0.60
Time in zone RT	%	3.62 ± 2.47	5.43 ± 2.90	0.70
Time in zone RB	%	2.59 ± 1.57	2.20 ± 1.17	0.86

Results are presented as the mean ± SEM and analyzed using a linear model. For detailed outline of the open field test, including a schematic overview of the arena, the percentage of animals able to stand and included in the test, representative heatmaps of cumulative tracking in the arena, and correlation between neuromotor score and birth weight, see Extended Data Table 1-1.

**Table 2:** Total brain and region weights in control and IGF-1 pigs at P5 and P9

	CON		IGF-1		*p*	
	P5	P9	P5	P9	P5	P9
Number of animals	17 (F/M, 6/11)	21 (F/M, 10/11)	18 (F/M, 8/10)	21 (F/M, 13/8)		
Total body weight (g)	1046.3 ± 58.75	1203.33 ± 65.89	994.17 ± 64.96	1285.81 ± 82.36	0.53	0.133
Total brain (g)	26.58 ± 0.40	30.04 ± 0.48	26.50 ± 0.44	30.23 ± 0.47	0.55	0.79
Total brain (%)	2.66 ± 0.14	2.63 ± 0.13	2.83 ± 0.16	2.52 ± 0.14	0.29	0.33
Cerebrum (g)	20.93 ± 0.30	24.44 ± 0.40	20.93 ± 0.35	24.64 ± 0.42	0.62	0.72
Cerebrum (%)	78.77 ± 0.53	81.36 ± 0.16	79.01 ± 0.36	81.46 ± 0.2	0.85	0.50
Cerebellum (g)	2.47 ± 0.07	2.72 ± 0.06	2.35 ± 0.05	2.75 ± 0.04	0.35	0.77
Cerebellum (%)	9.27 ± 0.21	9.04 ± 0.08	8.86 ± 0.07	9.1 ± 0.12	0.14	0.77
Hippocampus (g)	0.44 ± 0.02	0.50 ± 0.01	0.46 ± 0.01	0.51 ± 0.01	0.33	0.36
Hippocampus (%)	1.67 ± 0.06	1.65 ± 0.04	1.76 ± 0.05	1.69 ± 0.03	0.40	0.37
Brainstem (g)	2.622 ± 0.1	2.88 ± 0.05	2.58 ± 0.06^*a*^	2.85 ± 0.05	0.42	0.46
Brainstem (%)	9.85 ± 0.32	9.60 ± 0.15	9.79 ± 0.20^*a*^	9.44 ± 0.11	0.48	0.23
Striatum (g)	0.24 ± 0.01	0.31 ± 0.01	0.23 ± 0.01	0.31 ± 0.01	0.21	0.58
Striatum (%)	0.90 ± 0.04	1.03 ± 0.03	0.86 ± 0.05	1.04 ± 0.03	0.15	0.59
Water content (%)	84.86 ± 0.08	85.20 ± 0.09	85.02 ± 0.07	85.02 ± 0.08	0.20	0.19
Brain/liver ratio	0.918 ± 0.057	0.640 ± 0.031	1 ± 0.07	0.65 ± 0.04	0.16	0.98

Relative values (%) are the weight of a brain region relative to the total brain weight or total brain weight relative to body weight. Results are presented as the mean ± SEM and were analyzed using a linear model for each time point. F, Female; M, male.

a *n* = 17.

### IGF1R is differentially expressed in the developing pig brain

**Figure 1.**
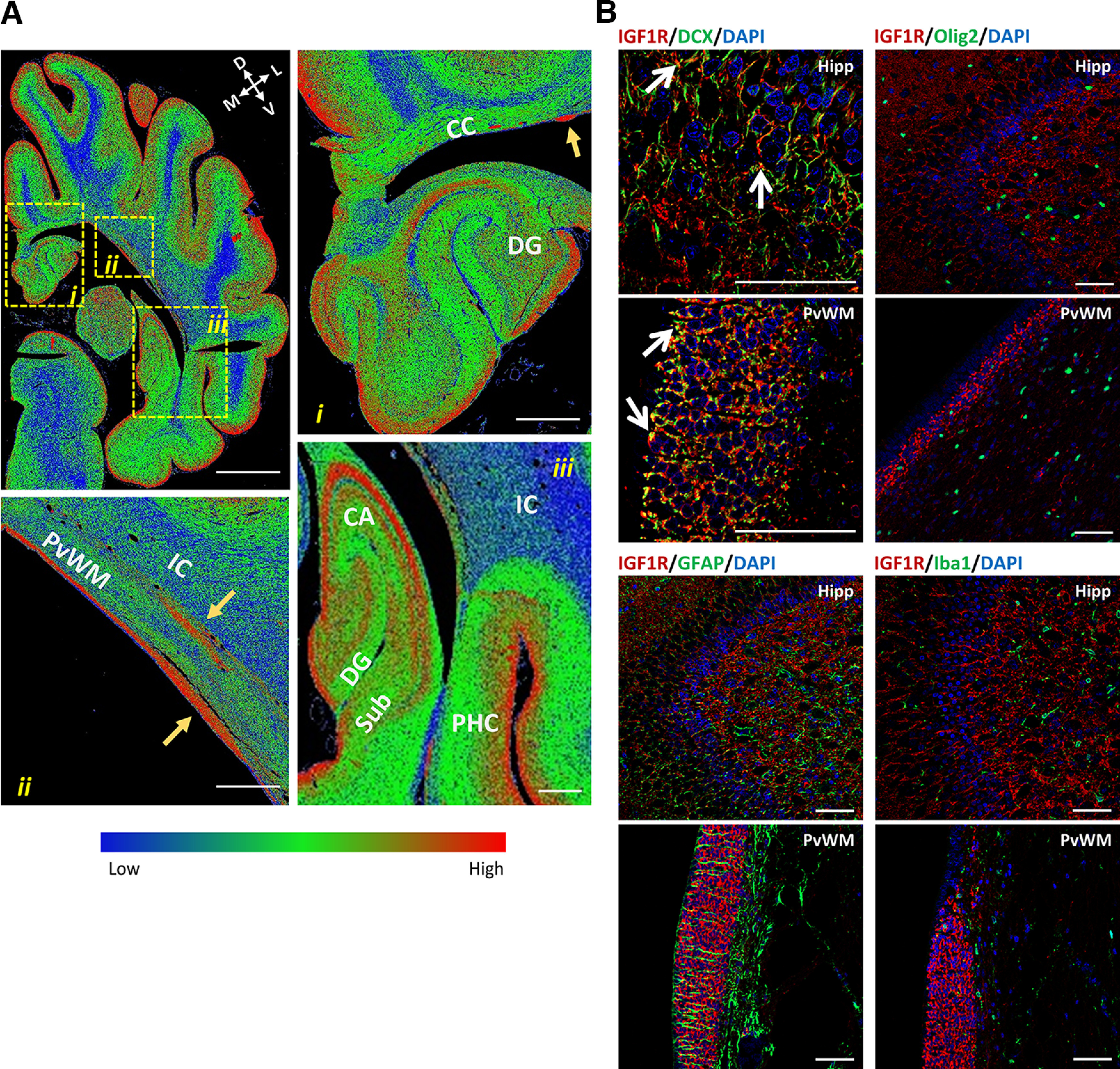
Expression of IGF1R in the developing pig brain. ***Ai–iii***, IGF1R immunoreactivity shown as density color-coded heatmaps from coronal sections of the right brain hemisphere at postnatal day 1, in the dorsal (***i***) and ventral (***iii***) hippocampus, and in PvWM (arrows; ***ii***). Blue–green–red colors correspond to low-, middle-, and high-density IGF1R immunoreactivity, respectively. D, Dorsal; V, ventral; M, medial; L, lateral. Scale bars: ***Ai–iii***, 5 mm; ***Ai–iii*** insets, 1 mm. CC, corpus callosum; PHC, perirhinal cortex; Sub, subiculum. Arrows (yellow) indicate clusters of cells with high levels of IGF1R expression. ***B***, Representative confocal microscopy images of the hippocampus (Hipp) and PvWM showing IGF1R (red) and DCX (green) fluorescence labeling, which is displayed as yellow when colocalized. Arrows (white) indicate areas with double staining. Double staining of IGF1R (red) with Olig2, GFAP, or Iba1 (all green) showed no overlapping labeling. Cell nuclei were counterstained with DAPI (blue). Scale bar, 50 μm. For details about primary and secondary antibodies used for labeling, see Extended Data Fig. 1-1. For additional images and single-channel images of IGF1R, DCX and Olig2 fluorescence labeling, see Extended Data Fig. 1-2.

First, the expression and localization of IGF1R in the brain were characterized using samples from newborn pigs (P1) and CON pigs from P9. IGF1R immunoreactivity (IR) of different intensities was widely present in the brains of P1 and P9 CON preterm pigs. Digital heatmaps of entire coronal sections showed high levels of IGF1R-IR (Fig. 1*A*) in the outer layers of the cerebral cortex, particularly in the parietotemporal cortex and entorhinal and perirhinal cortices, in clusters of cells within periventricular WM (PvWM; Fig. 1*Aii*), and in the pyramidal cell layers of the dorsal and ventral hippocampus and the dentate gyrus (DG; Fig. 1*Ai*,*iii*). Confocal microscopy images confirmed the higher expression of IGF1R in the hippocampus, PvWM, and cortex than in the white matter (Extended Data Fig. 1-2*C*). Relatively low levels of IGF1R were detected in major white matter tracts, such as the internal capsule (IC) and subcortical white matter [intragyral WM (IGWM); Fig. 1*A*]. To characterize whether IGF1R expression occurs in specific cell populations, costaining of IGF1R with DCX (immature neurons), Olig2 (pan-oligodendrocyte), GFAP (mature astrocyte marker), or Iba1 (microglia marker) was performed in the brains of P1 preterm pigs. In addition, DCX and IGF1R colabeling was characterized at P9. IGF1R cellular colocalization with DCX was detected in the hippocampus and in clusters of immature neurons located within the periventricular layer, both at P1 and P9 (Fig. 1*B*, Extended Data Fig. 1-2*A*,*B*). Cellular colabeling was detected both in the outer cell membrane and in distal processes. When coexpressed in the same structural brain regions, cellular colocalization was not observed for IGF1R-IR with Olig2, GFAP, or Iba1 labeling (Fig. 1*B*, Extended Data Fig. 1-2*C*). The widespread expression of IGF1R in the hippocampus and cortical parenchyma (Fig. 1*A*, Extended Data Fig. 1-2*C*) suggests a role of IGF-1 in the developing preterm pig brain.

10.1523/ENEURO.0430-22.2023.f1-1 Figure 1-1 List of primary and secondary antibodies, dilutions, and supplier information. Download Figure 1-1, DOCX file.

**Figure 2.**
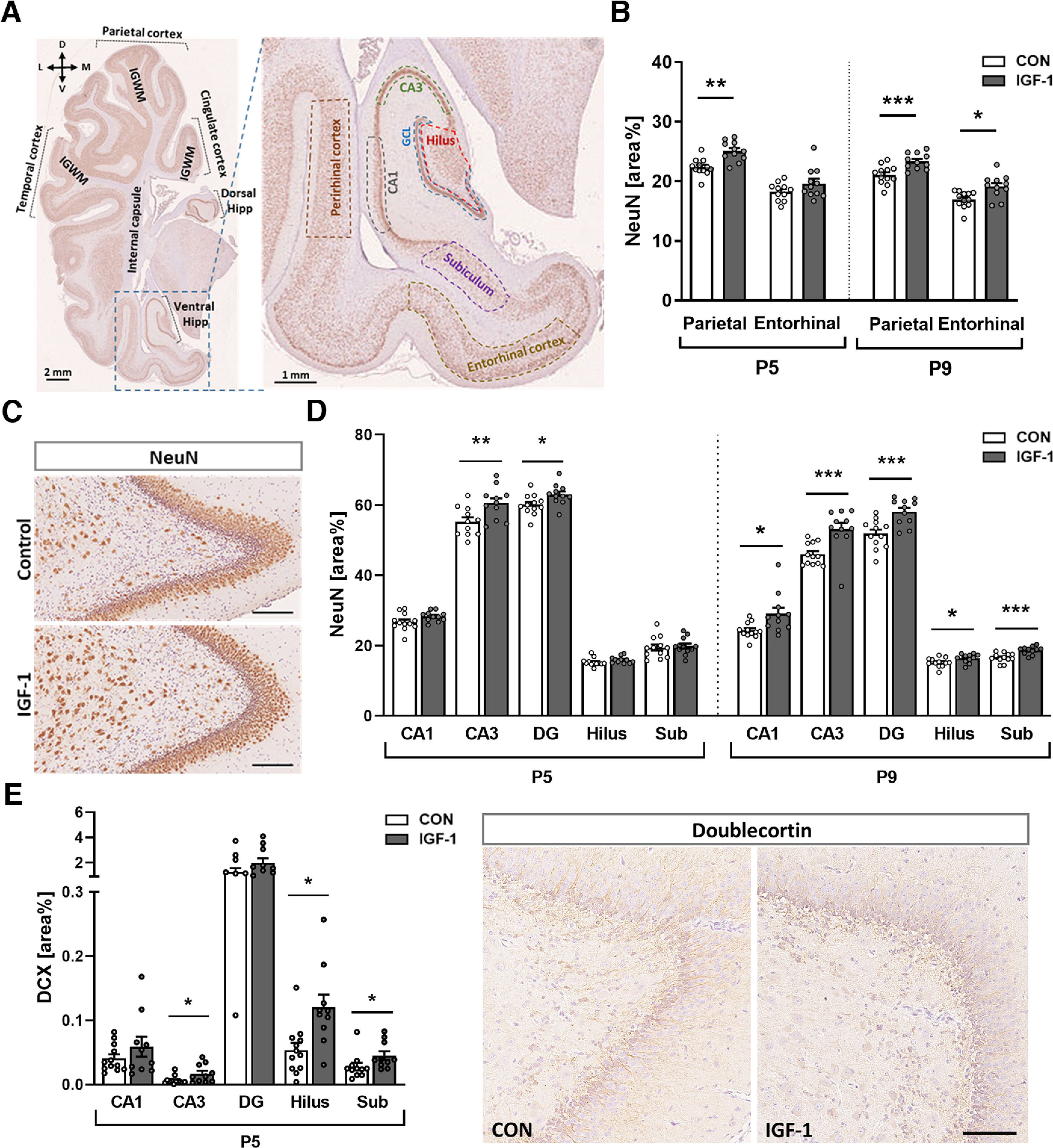
Exposure to IGF-1 promotes neuronal maturation in a spatiotemporal manner. ***A***, Representative image of NeuN/hematoxylin-labeled section illustrating the selected regions of interest analyzed in the study, including IGWM, cingulate, parietal, temporal, and perirhinal cortexes. D, Dorsal; V, ventral, M; medial, L; lateral. Insert, Enlarged ventral hippocampal area with the following delineated subregions selected for analysis: perirhinal cortex, CA1, CA3, granular cell layer (GCL), and hilus. The subiculum separates the hippocampus from the entorhinal cortex. ***B***, Quantification of the intensity of NeuN-IR in cortical subregions at P5 and P9, respectively. ***C***, Representative images of NeuN/hematoxylin-labeled dentate gyrus on P9 from pigs treated with vehicle (CON) or IGF-1. Scale bar, 200 μm. ***D***, Quantification of the intensity of NeuN-IR in hippocampal subregions (average of ventral and dorsal) at P5 and P9. DG, Granular cell layer of DG; Sub, subiculum. ***E***, Left, Quantification of the intensity of DCX-IR in subregions of the ventral hippocampus at P5. *n* = 11–12/age and treatment group. Right, Representative images of DCX/hematoxylin-labeled dentate gyrus on P5 from pigs treated with vehicle (CON) or IGF-1. Scale bar, 100 μm. Values are presented as the mean ± SEM. All data were analyzed using a linear model for each time point. Statistically significant effects of treatment are shown as follows: *p <* *0.05, **p <* *0.01, ***p <* *0.001.

10.1523/ENEURO.0430-22.2023.f1-2 Figure 1-2 Representative confocal microscopy images illustrating the expression of IGF1R. ***A***, Images illustrate the localization of IGF1R (red) and DCX (green) labeling in the hippocampus at birth (P1). ***Bi–iv***, At P9, the colocalization (yellow) of IGF1R (green) and DCX (red) was still observed in hippocampal CA3 (***Bii***) and neuronal progenitor cell clusters (***Bi***,***iii***), while faint colocalization was observed in cellular clusters located within PvWM (***Biv***). ***C***, Representative confocal microscope images showing IGF1R (red) expression in the hippocampus (Hipp), PvWM, WM, and cortical regions. Cell nuclei were counterstained with DAPI (blue). Scale bar, 10 μm. Download Figure 1-2, TIF file.

10.1523/ENEURO.0430-22.2023.tab1-1 Table 1-1 Open-field test at postnatal day 7. ***A***, Location and annotation of virtual zones in the open field arena, LT, RT, RB, and LB. ***B***, The number of animals able to stand at postnatal day 7 was not different between the CON (*n* = 16) and IGF-1 groups (*n* = 18). ***C***, To illustrate the cumulative tracks of pigs in the arena over 4 min for the IGF-1 and control groups, two representative open field heatmaps are shown for each group. Blue to red indicates low to high occupancy. ***D***, Correlation between birth weight and neuromotor score at postnatal day 7. Correlation data were analyzed by Pearson *R* correlation analysis, *p *<* *0.05 is considered statistically significant. Download Table 1-1, TIF file.

### IGF-1 treatment promoted neuronal maturation in a spatiotemporal manner

To measure whether systemic treatment with IGF-1 influences the neuronal cell population in the developing pig brain, the number of mature neurons (NeuN^+^ cells) and total area of NeuN-IR were quantified in the parietal and entorhinal cortex, subiculum, and different subregions of the dorsal and ventral hippocampus of control and IGF-1-treated pigs at P5 and P9 (Fig. 2*A*). The number of NeuN^+^ neurons in the total cortical area was not different between groups at P5 (CON, 71.49 ± 3.32; vs IGF-1, 71.55 ± 3.31; *p = *0.775) and P9 (CON, 63.16 ± 3.83; vs IGF-1, 65.07 ± 3.31; *p = *0.212), while in the hippocampus it was increased in IGF-1 pigs at P9 (CON, 46.4 ± 5.5; vs IGF-1, 47.5 ± 3.5; *p *=* *0.025). However, the area of NeuN-IR labeling was significantly higher in cortical and entire hippocampal areas at P5 in the IGF-1 group (cortex, *p *=* *0.038; hippocampus, *p *=* *0.049) and within the total cortical region at P9 (CON, 19.1 ± 1.2; vs IGF-1, 21.5 ± 1.5; *p *=* *0.0008). Quantification of NeuN-IR within different cortical and hippocampal subareas showed time-dependent and region-dependent changes. In the cortex, the mean NeuN-IR was significantly higher in the parietal cortex at P5 (*p *=* *0.001) and in both the parietal cortex (*p *=* *0.0004) and entorhinal cortex at P9 in the IGF-1 group (*p *=* *0.02; Fig. 2*B*). In the hippocampus, the IGF-1 group also had higher NeuN-IR in cornu ammonis 3 (CA3; *p *=* *0.002) and in the area of granular cells of the DG (*p *=* *0.03) at P5 and P9 (CA3, *p *=* *0.0004; DG, *p *=* *0.0009; Fig. 2*C*,*D*). Furthermore, in the subiculum and other hippocampal subregions, such as the CA1 and hilus, the area of NeuN-IR at P9 was also significantly higher in the IGF-1 group (CA1, *p *=* *0.022; hilus, *p *=* *0.043; subiculum, *p *=* *0.0005; Fig. 2*D*). These findings indicate that treatment with IGF-1 in preterm pigs accelerates the maturation of neurons in a region-specific manner during early postnatal development.

Quantification of the area of DCX^+^-IR in the same ROIs as for NeuN labeling showed a significant increase in the IGF-1 group only at P5 and was limited to the subiculum (*p *=* *0.01) and ventral hippocampus (*p *=* *0.047), specifically to different subfields of the ventral hippocampus, including the CA3 (*p *=* *0.048) and hilus (*p *=* *0.02; Fig. 2*E*).

### Synaptophysin expression is moderately affected by IGF-1 treatment at P9

**Figure 3.**
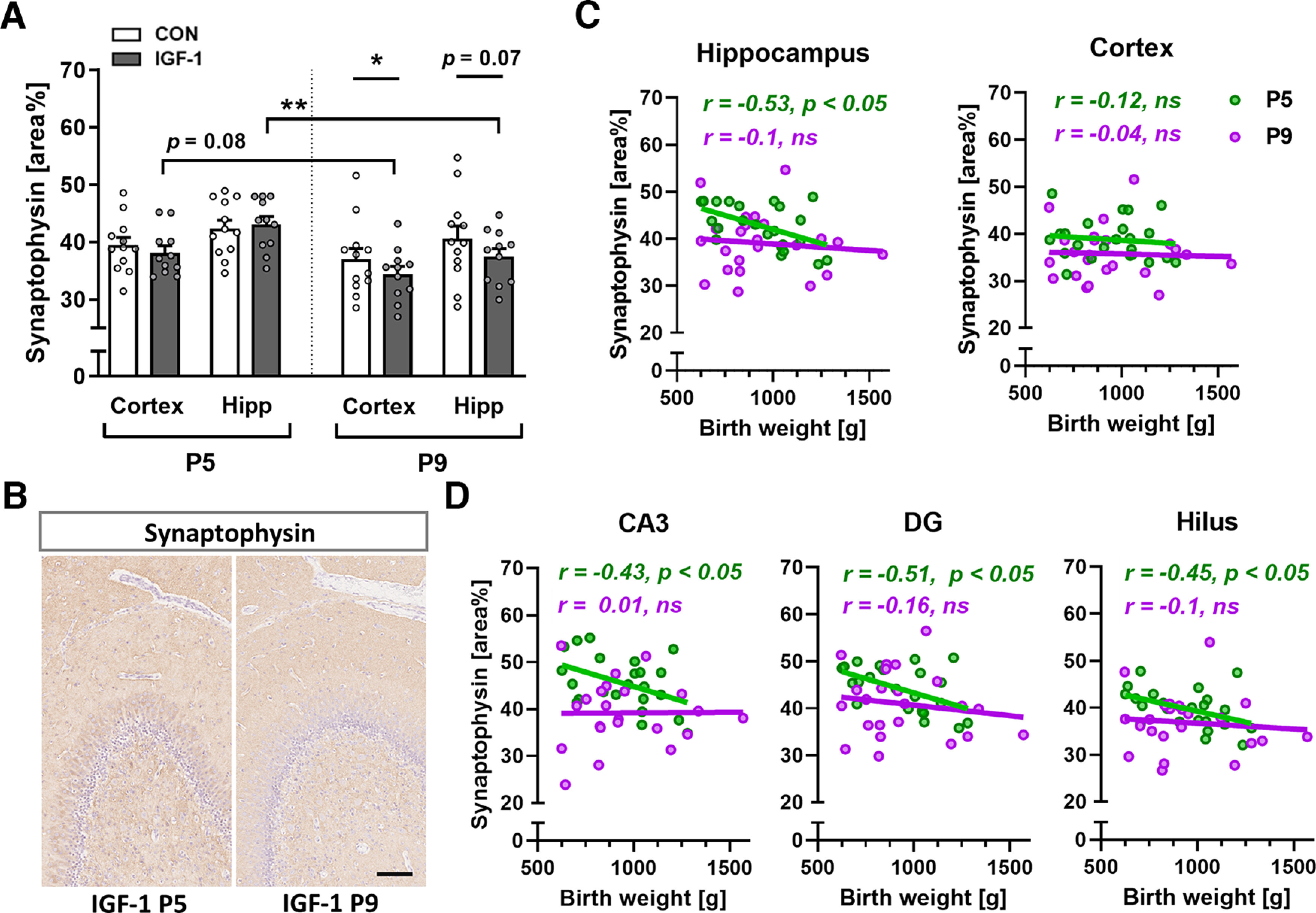
IGF-1 treatment is significantly associated with decreased synaptophysin expression on P9, when synaptophysin levels are no longer correlated with birth weight. ***A***, Quantification of synaptophysin-IR in the total cortex area (average of temporal, cingulate, parietal, and entorhinal cortex) and hippocampus proper (Hipp; average of dorsal and ventral CA1, CA3 (plus pyramidal layer), dentate gyrus molecular layer plus subgranular zone, hilus, and ventral subiculum quantification) on P5 and P9. *n* = 11–12/age and treatment group. Values are presented as the mean ± SEM. Data were analyzed using a linear model for each time point and for each treatment group. Statistically significant effects of treatment or time are shown as follows: *p <* *0.05; **p <* *0.01. ***B***, Representative images of synaptophysin/hematoxylin-labeled dentate gyrus on P5 and P9 from pigs treated with IGF-1. Scale bar, 100 μm. ***C***, ***D***, Correlation between birth weight and synaptophysin-IR on P5 (green) and P9 (purple) in the cortex and hippocampus proper (***C***), and in hippocampal subregions CA3, granular cell layer of DG, and hilus (***D***); data from hippocampal regions are the average of dorsal and ventral quantifications. Correlation data were analyzed by Pearson *R* correlation analysis; *p *<* *0.05 is considered statistically significant. ns, Nonsignificant.

Next, we sought to examine the area of Syn-IR, an integral membrane protein of synaptic vesicles, commonly used as a marker of synaptic density (Masliah et al., 1990). Compared with P5, an overall tendency of decreased Syn-IR was observed at P9 in cortical areas of both groups and significantly in hippocampal regions of the IGF-1 group (*p *=* *0.009; Fig. 3*A–C*). Compared with CON, treatment with IGF-1 had no effect on Syn-IR at P5 in all analyzed regions, including the parietal and entorhinal cortexes and hippocampal subfields, whereas at P9, it was decreased in the IGF-1 group in the total cortical areas (*p *=* *0.032) and showed a tendency to lower IR in the hippocampus (*p *=* *0.07; Fig. 3*A*). Interestingly, we observed a significant negative correlation between birth weight and Syn-IR in the entire hippocampus (*r* = −0.53; *p *=* *0.01) but not in the total cortical region at P5 (Fig. 3*C*). Specifically, at P5, a higher birth weight significantly correlated with lower Syn-IR in the CA3, DG and hilar regions of the hippocampus, while at P9, this correlation disappeared (Fig. 3*D*).

### IGF-1 treatment promoted oligodendrocyte development and myelination in a spatiotemporal manner

**Figure 4.**
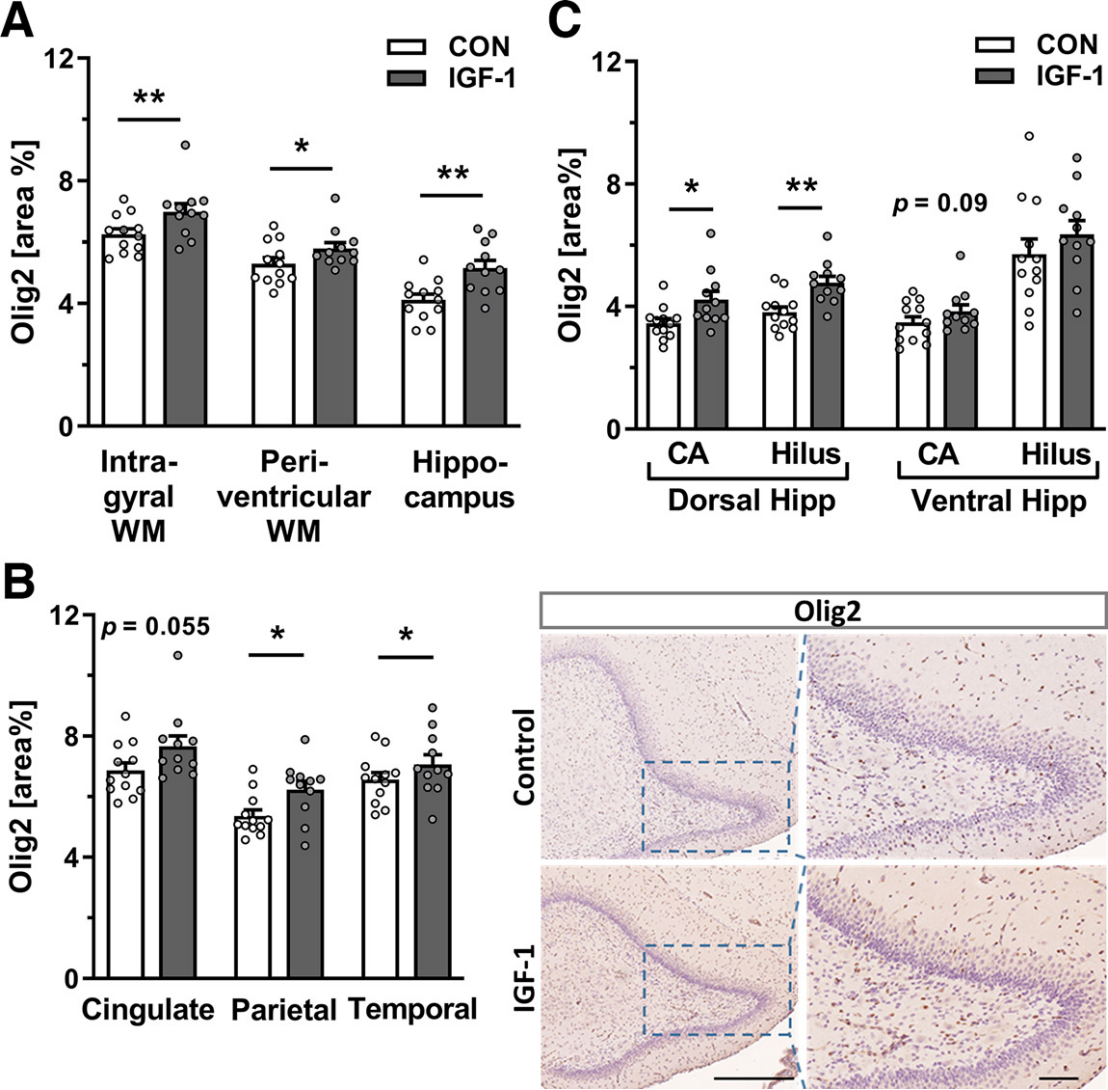
Continuous exposure to systemic IGF-1 promotes oligodendrocyte development. ***A–C***, Quantification of the area of Olig2^+^-IR on P5 in IGWM (average of temporal, cingulate, and parietal subcortical tracts), PvWM, hippocampus proper (average of dorsal and ventral CA and hilus quantification; ***A***), in different WM subcortical tracts (***B***, left), and in separate dorsal and ventral hippocampal total CA and hilar region at P5 (***C***). *n* = 11–12/treatment group. Data are given as the mean ± SEM. All data were analyzed using a linear model for each time point; statistically significant effects of treatment are shown as *p <* *0.05, **p <* *0.01. ***B***, Right, Representative images of Olig2 labeling in the dentate gyrus of control and IGF-1-treated pigs at P5. Scale bars: left, 500 μm; right, 100 μm. Sections were counterstained with hematoxylin. For the effect of IGF-1 treatment on Olig2^+^ cell number and on the level of Olig2 on postnatal day 9, see Extended Data Figure 4-1.

To determine whether systemic treatment with IGF-1 during the early perinatal period influences myelination, the total number of oligodendrocytes (Olig2^+^ cells) was counted, and the immunoreactive area of Olig2 and MBP were quantified in ROIs of WM tracts, including IGWM, IC, PvWM projections, and hippocampal subregions of preterm pigs at P5 and P9. Compared with CON, IGF-1 treatment had a significant effect on the number of Olig2^+^ cells in the IGWM (*p *=* *0.022) and hilar region of the hippocampus (*p *=* *0.038; Extended Data Fig. 4-1*A*) at P5. Likewise, the area of Olig2-IR was also higher in total IGWM (*p *=* *0.003) and PvWM (*p *=* *0.048) and in the hippocampus (*p *=* *0.009) of IGF-1-treated pigs at P5 (Fig. 4*A*), particularly in the total CA area (*p *=* *0.037). When total IGWM was subdivided into (1) cingulate, (2) parietal, and (3) temporal subregions, the mean area of Olig2^+^-IR was significantly higher in the parietal and temporal subcortical areas for the IGF-1 group (*p *=* *0.016 and *p *=* *0.014, respectively; Fig. 4*B*). Furthermore, the increase in Olig2-IR area in the hippocampus (*p *=* *0.009; Fig. 4*A*) was driven by the dorsal part of the hippocampal formation, which showed significantly higher values of Olig2-IR in the IGF-1 group in both dorsal CA and hilus fields (*p *=* *0.03 and *p *=* *0.005, respectively; Fig. 4*C*). While these changes were observed in the first postnatal week (P5), at P9 no difference was found between the CON and IGF-1 groups for the number of Olig2^+^ cells and the level of the Olig2-IR area in these regions, with the exception of Olig2^+^ cell numbers increasing in the ventral CA field of IGF-1 pigs (*p *=* *0.046; Extended Data Fig. 4-1*C*).

10.1523/ENEURO.0430-22.2023.f4-1 Figure 4-1 ***A***, Quantification of the number of Olig2^+^ cells in the IGWM (average of temporal, cingulate, and parietal subcortical tracts), PvWM, CA, and hilar region (both average of respective dorsal and ventral hippocampal quantifications) of the hippocampus at postnatal day 5. CON, *n* = 12; IGF-1, *n* = 11. ***B***, ***C***, Quantification of the area of Olig2^+^-IR (***B***) and the number of Olig2^+^ cells (***C***) on postnatal day 9 in the PvWM, IGWM (average of temporal, cingulate, and parietal subcortical tracts), dorsal and ventral CA, and hilus of the hippocampus (Hipp). All results are expressed as the mean ± SEM. All data were analyzed using a linear model for each time point; statistically significant effects of treatment are shown as *p <* *0.05. Download Figure 4-1, TIF file.

**Figure 5.**
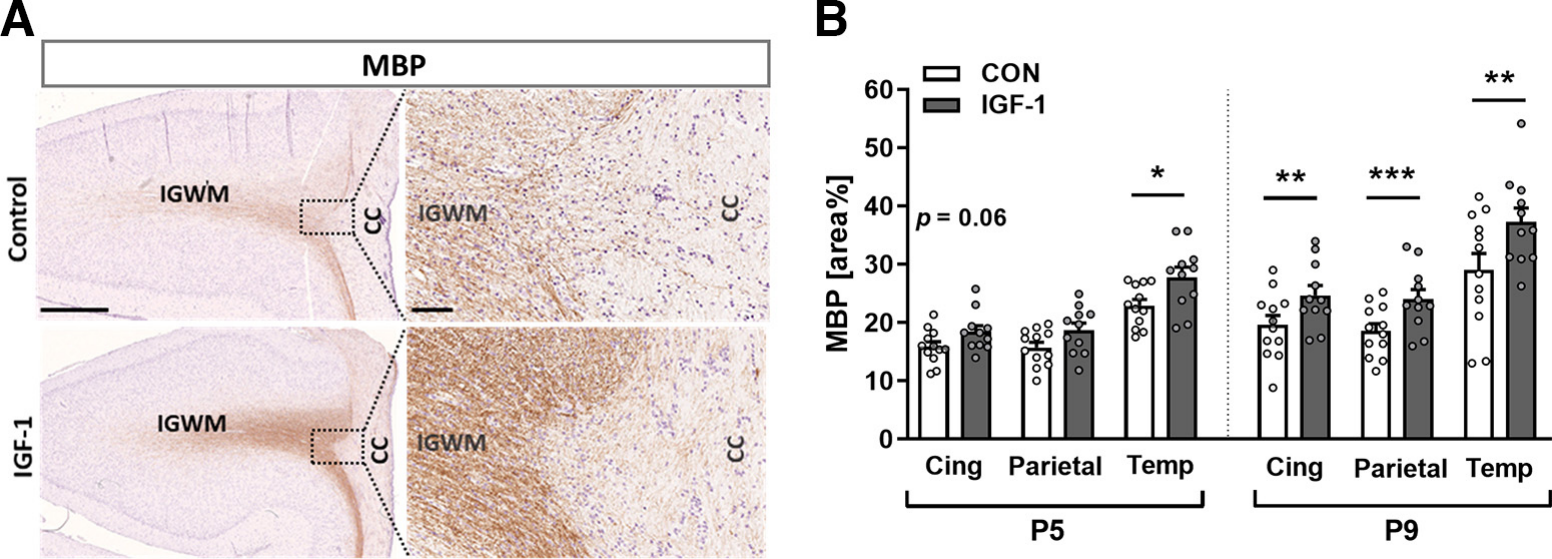
Treatment with IGF-1 promotes myelination of fiber tracts in preterm pigs. ***A***, Representative images of the posterior cingulate cortex on P9 at low magnification (left; scale bar, 1 mm) and high magnification (right; scale bar, 100 μm), immunostained for MBP and counterstained with hematoxylin. Preterm-born pigs were treated with either vehicle (Control; top panel) or IGF-1 (bottom panel). CC, Corpus callosum. ***B***, Quantification of myelination in three intragyral white matter regions: cingulate (Cing), parietal and temporal (temperature) cortices on P5 and P9. CON, *n* = 12; IGF-1, *n* = 11. Data are given as the mean ± SEM. All data were analyzed using a linear model for each time point. Statistically significant effects of treatment are shown as *p <* *0.05, **p <* *0.01, ***p <* *0.001.

Analysis of MBP-IR in the brains of preterm pigs showed progressive myelination processes from P5 to P9 for most of the analyzed regions in both the CON and IGF-1 groups. Compared with CON pigs, IGF-1 pigs had a significantly higher MBP-IR area in the total IGWM region, both at P5 (CON, 18.17 ± 0.69; vs IGF-1, 21.68 ± 1.07; *p *=* *0.019) and P9 (CON, 22.82 ± 1.77; vs IGF-1, 28.35 ± 1.62; *p *=* *0.002). Detailed analysis of MBP-IR in three predetermined areas of IGWM located within the cingulate gyrus and parietal and temporal cortices showed region-dependent and time-dependent effects of IGF-1 treatment on myelination. Specifically, at P5 only temporal IGWM had significantly higher MBP-IR (*p *=* *0.027), whereas at P9, all three analyzed intragyral subfields had higher MBP-IR in the IGF-1 group (*p *=* *0.008 for temporal, *p *=* *0.0003 for parietal, and *p *=* *0.008 for cingulate IGWM; Fig. 5*A*,*B*). No differences in MBP-IR between the CON and IGF-1 groups were observed in the hippocampal subregions at P5 or P9. Together, data on both Olig2-IR and MBP-IR indicate that systemic treatment with IGF-1 accelerated ongoing myelination and promoted the maturation of oligodendrocytes in a region-specific manner.

### Effects of IGF-1 on hippocampal gene expression, brain protein synthesis, and microglia

**Figure 6.**
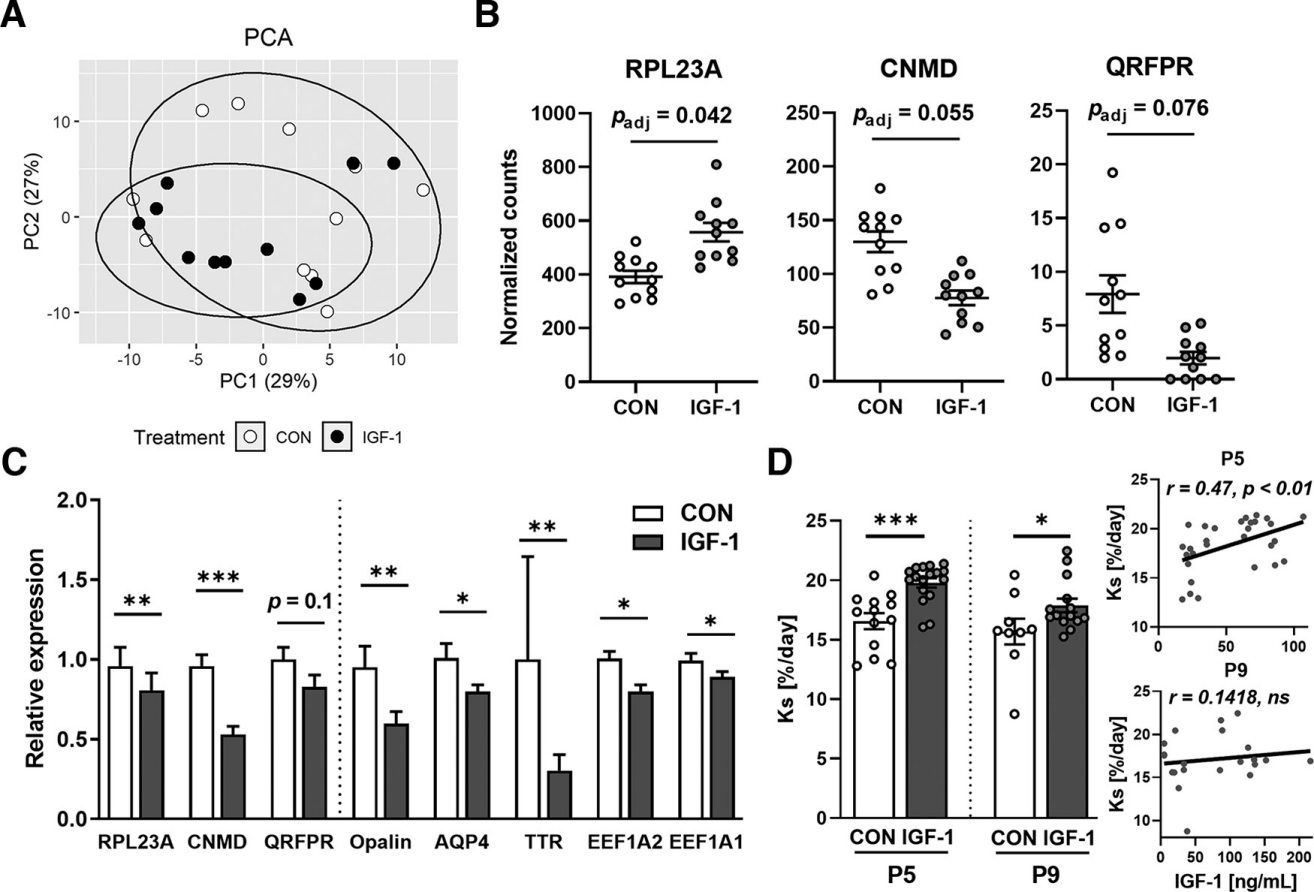
Hippocampal transcriptome profile in response to IGF-1 treatment. ***A***, PCA plot demonstrating marginal segregation of hippocampal gene expression in the CON and IGF-1 groups at P5. ***B***, Boxplot of three significant differentially expressed genes between CON and IGF-1. *n* = 11/treatment group. ***C***, Relative expression (fold change compared with CON) of selected genes in the hippocampus of P5 pigs analyzed by qPCR. *n* = 11–13/treatment group. ***D***, Left, Ks values (percentage of protein mass synthesized per day) in the cerebellum measured in the CON and IGF-1 groups at P5 and P9. ***D***, Right, Correlation between Ks and plasma IGF-1 levels at P5 and P9. In ***B***–***D***, results are presented as the mean ± SEM. Data were analyzed using a principal component analysis (***A***), DESeq2 (***B***), using a linear model for each time point (***C***, ***D***, right), or Pearson *R* correlation (***D***, left), a Benjamini–Hochberg correction was applied in ***B***; in ***C*** and ***D***, statistically significant effects of treatment are shown as *p <* *0.05, **p <* *0.01, ***p <* *0.001. ns, Nonsignificant. For qPCR primer list, see Extended Data Figure 6-1. For detailed RNA-sequencing results, see Extended Data Figure 6-2. For the effect of IGF-1 treatment on the expression of genes related to IGF-1 signaling and gene expression on P9, see Extended Data Figure 6-3*A–C*. For details on Iba-1 labeling, see Extended Data Figure 6-3*D–F*.

The RNA-seq results showed that among 16,681 pig genes with ascertainable gene symbols, the individual expression level of 670 genes differed between groups (*p *<* *0.05; Extended Data Fig. 6-2). Principal component analysis (PCA) showed no obvious separation, indicating similar hippocampal gene expression between IGF-1 and CON pigs (Fig. 6*A*). Using DESeq2 with an adjusted *p *<* *0.1 threshold, only three genes were identified as DEGs, including upregulation of *RPL23A*, encoding ribosomal protein L23a, a component of the large ribonucleoprotein complex responsible for protein synthesis, and downregulation of *CNMD* and *QRFPR,* encoding chondromodulin and pyroglutamylated RFamide peptide receptor, respectively (Fig. 6*B*, Extended Data Fig. 6-2). Downregulated *CNMD* expression was confirmed by qPCR (*p *<* *0.001 between groups; *r* = 0.74, *p *<* *0.001 for RNA-seq to qPCR correlation), and *QRFPR* expression tended to be reduced (*p *=* *0.1; Fig. 6*C*). In addition to the RNA-seq results (Extended Data Fig. 6-2), the expression of selected genes related to glial and neuronal maturation was measured by qPCR, including *Opalin* (*p *<* *0.001), *AQP4* (*p *<* *0.05), *TTR* (*p *<* *0.01), and *EEF1A2* (*p *<* *0.05; Fig. 6*C*). These changes in expression did not persist until P9 (Extended Data Fig. 6-3*A*). Likewise, for genes related to IGF-1 signaling, expression (as measured by qPCR) was not affected by IGF-1 treatment for *IGF1*, *IGF2*, *IGFBP3*, *IGF1R*, *IGF2R*, and the *IRS1* gene (encoding InR substrate 1) at P5 (Extended Data Fig. 6-3*B*) or P9 (Extended Data Fig. 6-3*C*).

10.1523/ENEURO.0430-22.2023.f6-1 Figure 6-1 List of primers for qPCR. F, Forward primer; R, reverse primer; ^¤^, primers for these genes were adopted from Pan et al. (2012). Download Figure 6-1, DOCX file.

10.1523/ENEURO.0430-22.2023.f6-2 Figure 6-2 DEGs between the control and IGF-1 groups at postnatal day 5 identified by RNA-seq analysis. Listed are all identified porcine genes with corresponding location, the (base 2) log of the fold change, the uncorrected *p* value and FDR-adjusted *p* value (*p*adj). Download Figure 6-2, XLS file.

10.1523/ENEURO.0430-22.2023.f6-3 Figure 6-3 ***A***, ***C***, Relative expression (fold change compared with CON) of selected genes (***A***) and genes related to IGF-1 signaling in the hippocampus on P9 analyzed by qPCR (***C***). *n* = 7–8/treatment group. ***B***, Relative expression (fold change compared to CON) of genes related to IGF-1 signaling in the hippocampus of P5 pigs analyzed by qPCR. *n* = 11–13/treatment group. ***D***, Representative images of Iba1/hematoxylin-labeled dentate gyrus on P5 from pigs treated with vehicle (CON) or IGF-1. Scale bars: ***D***, 100 μm; inset, 10 μm. ***E***, ***F***, Quantification of the area of Iba1-IR on P5 (***E***) and P9 (***F***) in the PvWM, cortex, dentate gyrus (dorsal + ventral), hilus (dorsal + ventral), and subiculum. All results are expressed as the mean ± SEM. All data were analyzed using a linear model for each time point. Download Figure 6-3, TIF file.

IGF-1 supplementation significantly increased protein synthesis in the brain, as indicated by 19% and 14% greater cerebellar Ks values in IGF-1 than in CON pigs at P5 and P9, respectively (Fig. 6*D*). The protein synthesis rates positively correlated with plasma IGF-1 levels at P5 (*r *=* *0.47, *p *=* *0.0099), but not at P9 (*r *=* *0.14, *p *=* *0.54; Fig. 6*D*).

Finally, we evaluated whether the area of Iba1-IR, representing microglial cells, was affected by IGF-1 treatment (Extended Data Fig. 6-3*D*). A tendency toward increased values was observed in the IGF-1 group in the dorsal DG at P5 (CON, 3.21 ± 0.13; vs IGF-1, 3.73 ± 0.2; *p *=* *0.061) and ventral DG at P9 (CON, 2.7 ± 0.32; vs IGF-1, 2.9 ± 0.26; *p *=* *0.07). No differences were detected between groups in the remaining hippocampal areas and all other analyzed ROIs, such as cortical areas, subiculum, IC, and PvWM at P5 or P9 (Extended Data Fig. 6-3*E*,*F*). Likewise, no differences between groups were detected for hippocampal expression of inflammation-related genes *IL6* (IGF-1 fold change: P5: 0.84 ± 0.05, *p *=* *0.25; P9: 0.79 ± 0.05, *p *=* *0.23); *IL10* (IGF-1 fold change: P5: 1.09 ± 0.10, *p *=* *0.63; P9: 1.98 ± 0.85, *p* = 0.24); and *S100A9* (IGF-1 fold change: P5: 0.87 ± 0.12, *p *=* *0.84; P9: 1.51 ± 1.06, *p *=* *0.46), and were analyzed with qPCR.

## Discussion

Preterm infants show reduced plasma IGF-1 levels for several weeks after birth, and this condition has been speculated to contribute to impaired brain development and a high risk of neurodevelopmental disorders (Hansen-Pupp et al., 2011, 2013; Hellström et al., 2016). Multicenter intervention studies are currently testing the effects of supplemental IGF-1/IGFBP3 in extremely preterm infants on respiratory outcomes and ROP (ClinicalTrials.gov Identifiers NCT03253263 and NCT01096784, respectively; Horsch et al., 2020). The possible effects of such treatment on premature brain growth and development are unknown. Using preterm pigs as a model, we observed that systemic injection of IGF-1 stimulated brain protein synthesis and promoted gray matter and white matter maturation in a spatiotemporal manner, but did not affect brain weight or motor skills in the early perinatal period.

Preterm pigs are a good proxy to explore human brain development, as their brain anatomy, developmental trajectory in the perinatal period (Dobbing and Sands, 1979), gray matter to white matter ratio (Zhang and Sejnowski, 2000), and region-dependent maturation processes are similar to those in humans (Ishibashi et al., 2012; Stinnett et al., 2017; Liu et al., 2021), although functional aspects of brain development (e.g., neuromuscular development and movement control) develop earlier in pigs versus infants. Additionally, preterm pigs have low circulating IGF-1 levels and immature immune, gut, and metabolic systems, making them sensitive to sepsis, hypoglycemia, and gut disorders such as NEC (Andersen et al., 2016; Nguyen et al., 2016; Holme Nielsen et al., 2018; Holgersen et al., 2020), for which they are a clinically relevant model of preterm infants.

Our results showed that the immature pig brain expressed IGF1R at the gene and protein levels, consistent with results from other newborn mammals, including humans (Liu et al., 2009). Immunoreactivity to IGF1R was detected throughout the brain with high densities in cortical regions and the hippocampus. At the cellular level, IGF1R staining was colocalized with DCX^+^ neurons, indicating that immature neurons are the primary target for systemic and/or locally produced IGF-1. The preterm pigs treated with vehicle had low plasma IGF-1 levels (27.4 ± 4.2 ng/ml at P5), which is in agreement with our previous reports (Andersen et al., 2016; Holgersen et al., 2020). While treatment with IGF-1, either intravenously for 5 d or subcutaneously for 9 d, increased plasma IGF-1 to the desired normophysiological level (70.9 ± 4.1 and 91.0 ± 11.8 ng/ml, respectively), this treatment length may have been insufficient to affect brain weight, motor skills, or water brain content (a surrogate marker of brain maturation; Holme Nielsen et al., 2018). The absence of an IGF-1 effect on early postnatal brain growth is consistent with observations that brain growth was not negatively affected until P40 in IGF-1 knock-out mice (Cheng et al., 2000). IGF-1 supplementation did not modulate hippocampal expression of *IGF1R* or *IGF1* or its downstream signaling elements at P5 and P9, indicating that physiological IGF-1 signaling in the brain was maintained. On the other hand, it cannot be excluded that the transient downregulation of *TTR* mRNA, encoding protein transthyretin, might negatively affect hippocampal IGF1R expression (Vieira et al., 2015), as it is known to act synergistically with IGF-1 on IGF1R activation (Vieira et al., 2016). Alternatively, the transient alterations in the expression of *IGF-1* and related genes might arise before P5, as it was recently shown for *IGF-1* expression in the choroid plexus of preterm rabbit pups treated with IGF-1/IGFBP3 complex (Gram et al., 2021). In this study, the greatest changes in gene expression were observed at 24 h and limited changes at 72 h after treatment start, suggesting an acute and transient effects of systemic IGF-1 on brain gene expression (Gram et al., 2021). Our RNA-seq data of the entire hippocampal structure at P5 showed marginal changes in gene expression, while IHC results at the same time point indicated clear region-dependent differences between groups. This mismatch between RNA-seq and IHC data could be explained by selected regions used for IHC, mRNA stability, regulation of translation by multitargeted small RNAs, and turnover rate of proteins (Schwanhäusser et al., 2011). Furthermore, our data are in agreement with known low correlation between transcriptomics and protein expression shown for many tissues including the brain (Bauernfeind and Babbitt, 2017).

Despite the lack of effect of IGF-1 supplementation on overall brain weight, IGF-1 treatment led to an increase in brain protein synthesis, suggesting high sensitivity of the neonatal brain to the anabolic effects of systemic IGF-1. The positive correlation between plasma IGF-1 levels and the rates of brain protein synthesis at P5 but not at P9 may suggest a developmental decline in IGF-1 responsiveness after preterm birth or reflect the differential response to continuous intra-arterial and intermittent subcutaneous IGF-1 administration. The data are in line with previously shown anabolic effects of systemic IGF-1 administration on other visceral organs and tissues, such as the heart, muscle, and spleen in neonatal pigs (Davis et al., 2002).

Treatment with IGF-1 affected the fraction of area immunoreactive to NeuN, a nuclear DNA binding protein expressed by postmitotic neurons (Mullen et al., 1992). The observed increase in area fraction of NeuN^+^-IR in both the cortex and hippocampus at P5 and at P9 suggests a time-dependent and region-dependent effect of IGF-1 on neuronal maturation, although the number of NeuN^+^ cells only increased in the hippocampus at P9 for the IGF-1 group and was similar to CON in the hippocampus at P5 and for both time points in the cortex. Previous studies have also shown limited correlation between NeuN immunoreactivity and neuron numbers when the physiological state has been affected, suggesting that altered NeuN expression is associated with maturation and stability of neurons rather than a direct estimation of neuron numbers (Unal-Cevik et al., 2004; Lavezzi et al., 2013; Duan et al., 2016). Our results are in agreement with a study in transgenic mice showing that IGF1R signaling regulated the maturation of newly formed neurons within the DG (Nieto-Estévez et al., 2016). In further support of our results, overexpression of IGF-1 increased the number of differentiated neurons in the cortex and DG before and after birth in mice (Popken et al., 2004). In the developing mouse brain, maturing neurons express elongation factor EEF1A2 in parallel with a decreasing expression of EEF1A1, which eventually disappears at P20 (Pan et al., 2004). We observed that IGF-1 may promote this transition in the preterm pig brain with an upregulation of *EEF1A2* in parallel with a decrease in *EEF1A1* expression at P5.

In addition to the differences observed in the hippocampal area and subiculum, we observed a higher area of NeuN-IR in the entorhinal cortex of IGF-1-treated P9 pigs, a region anatomically and functionally connected to the hippocampus and implicated in memory functions (Bellmund et al., 2019; Ronaghi et al., 2019). In humans, reduced thickness of the entorhinal cortex is correlated with lower cognitive scores in adolescents born very preterm (Skranes et al., 2012). In both pigs and humans, the entorhinal cortex develops during the second trimester (Liu et al., 2021). Additionally, in rodents, the entorhinal–hippocampal connection is formed in the fetus, and its circuits mature in the early neonatal period in region-dependent sequential waves (Deng et al., 2006; Donato et al., 2017; Valeeva et al., 2019). In preterm pigs, IGF-1 supplementation appeared to affect both the entorhinal cortex and hippocampal areas, particularly in CA1 and CA3, which are known to receive direct projections from the entorhinal cortex. This indicates that systemic IGF-1 can modulate the maturation of the entire entorhinal–hippocampal network, likely supporting advanced cognitive functions (Donato et al., 2017). The possibility that longer-term IGF-1 supplementation supports cognition and memory in preterm pigs remains to be investigated.

In mammalian brain development, neural circuits go through an initial rapid increase in the number of synapses followed by a gradual decrease by selective elimination via microglia-mediated pruning (Paolicelli et al., 2011). This sculpturing process, described in many regions of the developing mammalian brain (Bourgeois et al., 1989; Anderson et al., 1995; Zehr et al., 2006; Petanjek et al., 2011), depends on gestational age at birth and appears to be critical for learning potential (Afroz et al., 2016). The timing of synapse elimination is region and species dependent, with synaptic density being highest in infants at 1–2 years of age and declining toward adulthood (Glantz et al., 2007; Paolicelli et al., 2011). In this study, we observed a slight decrease in the area of immunoreactivity for the presynaptic marker synaptophysin over time in cortical areas and the hippocampus in both groups, potentially reflecting spine ramification over time or differences in IGF-1 administration for the P5 (intra-arterial) and P9 (subcutaneous) end points, although treated pigs at both time points had IGF-1 plasma levels within physiological relevant levels. Treatment with IGF-1 clearly promoted this developmental process in the cortex and with a tendency in the hippocampus at P9. The negative correlation between birth weight and Syn^+^-IR in the hippocampal regions at P5 (but not at P9) could suggest that the IGF-1-associated decrease in Syn^+^-IR seen at P9 (but not at P5) changes this correlation; thus, IGF-1 treatment overturns the effect of birth weight. However, the opposite might be true, and the levels of Syn^+^-IR could also be dependent on *in utero* growth and development, thus interfering with IGF-1 treatment effects on synaptic density on P5. In fetal lambs, dendritic tree density decreases with gestational age toward term, as estimated by MAP2 immunoreactivity (Czikk et al., 2015), but apart from this, little is known about neural circuit formation in larger animals.

Preterm infants are highly susceptible to cerebral white matter injury (Back, 2017). In contrast to oligodendrocyte progenitors, differentiating preoligodendrocytes are especially vulnerable to hypoxic and inflammatory insults, which may induce differentiation arrest and myelination failure (Back et al., 2002). IGF-1 transiently enhanced the number and area of Olig2^+^-IR at P5, which was followed by increased MBP-IR at P9 in subcortical regions, indicating a maturation effect on oligodendrocytes. Our observations are in agreement with results from transgenic mice, showing enhanced differentiation and maturation of oligodendrocytes overexpressing IGF-1 (Ye et al., 1995). Conversely, deletion of IGF-1 (Ye et al., 2002) or oligodendrocyte-specific conditional ablation of IGF1R inhibited myelination (Zeger et al., 2007).

In conclusion, our results show that systemic IGF-1 supplementation, within normal physiological levels, enhances gray and white matter development in the immediate postnatal period in preterm pigs. Specifically, our data suggest that IGF-1 targets immature neurons and promotes the expression of genes associated with the differentiation of neurons, which leads to an increased area and number of mature neurons in cortical and hippocampal regions. Furthermore, our data also suggest that IGF-1 promotes subcortical oligodendrocyte maturation, resulting in increased myelination. The effects were spatiotemporally dependent, thus reflecting the dynamic nature of neonatal brain development. It remains to be shown whether supplemental IGF-1 will also enhance motor skills and total brain volume later in life or following more prolonged IGF-1 administration. Our results indicate the clinical significance of IGF-1 supplementation for brain development in hospitalized preterm infants, but more studies are needed.

## Synthesis

Reviewing Editor: Michael Michaelides, NIDA-NIH Decisions are customarily a result of the Reviewing Editor and the peer reviewers coming together and discussing their recommendations until a consensus is reached. When revisions are invited, a fact-based synthesis statement explaining their decision and outlining what is needed to prepare a revision will be listed below. The following reviewer(s) agreed to reveal their identity: Hoda Gebril, Adrienne Antonson.

Reviewer 1

In this study, authors investigated the effects of supplemental IGF-1 on motor function and regional and cellular brain development. For this, authors used cesarean delivered preterm pigs as a model of preterm infants. They showed that the IGF-1 receptor was widely distributed in the brain and largely coexisted with immature neurons. Region-specific quantification of IHC labeling showed that IGF-1 treatment promoted neuronal differentiation, increased subcortical myelination and attenuated synaptogenesis in a region- and time-dependent manner. Also, the expression levels of genes involved in neuronal and oligodendrocyte maturation, angiogenic and transport functions were altered. Cerebellar protein synthesis was increased at Day 5 and Day 9 after IGF-1 treatment. In contrast, treatment had no effect on Iba1-positive microglia or regional brain weights and did not affect motor development or the expression of genes related to IGF-1 signaling.

Overall, this study provides further understanding for the importance of supplemental IGF-1 at early developmental stages in preterm infants. Nevertheless, long-term studies are needed to confirm the beneficial effect of IGF-1 supplementation on motor and neurological functions.

The manuscript is well written, and illustrations (including tables and figures) are convincing. However, to further improve this manuscript, I suggest the authors consider the following comments:

**Minor concerns**

1) In page 5, first paragraph: the anabolic and neuroactive effect of IGF-1in brain should be discussed in detail and specified.

2) In page 5, the following sentence is hard to follow:

“Previous studies have shown that preterm pigs have low plasma IGF-1 levels, thus resembling preterm neonates, and treatment with exogenous IGF-1 tended to reduce the incidence and severity of NEC”

3) In page 8, methods of tissue collection: It is unclear how the brain water fraction is estimated. If the method is to dry tissue to a specific weight, then this may cause over-dryness to some tissues and lead to overestimating water content in some samples.

4) In figure 1: the expression of DCX, GFAP, Olig2, and Iba-1 is hard to see. It is well established that all these markers are expressed well in hippocampus at this developmental stage. Although some single-channel images are provided in the extended figures, a better stain quality for these markers is crucial. I suggest adding single-channel images for each marker in figure 1. Also, adding arrows pointing toward colocalization would be helpful.

5) In figures 2-5: presenting cell count as cells [area%], is hard to understand. Indeed, the explanation in the methods section for area% needs to be clarified. A small ROI would have less number of cells than a big ROI in a test group which would lead to false positive results. There should be some standardization in all cell counting methods.

6) It would be helpful to include statistical analysis used for each graph in figure legend. Why there is extra data processing as stated in statistical analysis such as log-transformation? Also, what statistical analysis used/if any in Prism?

Reviewer 2

Major revisions:

Two different IGF-1 administration protocols are utilized in this study. The authors should describe their reasoning for selecting these two delivery modes and durations, and indicate how they compare to current clinical protocols (i.e., clinical trials NCT03253263 and NCT01096784).

The authors need to include an explanation for why expression of genes related to IGF-1 signaling were not altered at either time point in their models. Likewise, they need to discuss why the RNAseq data indicate no changes in gene expression (after adjusting for multiple comparisons), but IHC and protein synthesis data indicate changes in protein expression and turnover across almost all brain regions and time points.

The authors claim that IGF1R is differentially expressed in the developing pig brain, but no methods are reported for how these conclusions were made, and no statistical comparisons appear to have been made across brain regions or ROIs. This is odd considering there are methods reported for quantification of immunoreactive fraction (area %) for all other markers within specific ROIs. This should be done for IGF1R staining as well and proper statistical comparisons between regions should be performed. Further, cellular colabeling is reported but no methods are listed for determining whether a particular cell (DCX+, Olig2+, GFAP+ or Iba1+) expresses IGF1R or not. Methods for acquiring density color-coded heat maps of IGF1R staining also needs to be described.

In the discussion, the authors conclude: “The positive correlation between plasma IGF-1 levels and rates of brain protein synthesis at PND5 but not at PND9 may suggest a developmental decline in IGF-1 responsiveness after preterm birth.” However, IGF1R expression was not measured at PND5 nor compared to expression at PND9 (for that matter, there are no statistical comparisons between PND1 and PND9). Thus, the data do no support a decline in responsiveness. Instead, they support a possible difference in the two IGF-1 treatment schemes (5 days i.a. vs. 9 days s.c.). Furthermore, non-significant correlation between plasma IGF-1 levels and protein synthesis at PND9 could be explained by high variance within either of these parameters, but this is difficult to interpret as the plasma data (and the correlation) are only summarized in the text, not graphically represented. It is very possible s.c. delivery of IGF-1 does not reach and/or remain in circulation as effectively as i.a. delivery, and thus does not enter the brain at comparable rates to i.a. delivery. Similarly, the authors state: “The negative correlation between birth weight and Syn+-ir in the hippocampal regions at PND5 (but not at PND9) suggests that the levels of Syn+-ir are dependent on in utero growth and development, thus interfering with IGF-1 effects on synaptic density.” Again, this is not necessarily supported by the data. Instead, there are no group differences in Syn+-ir at PND5, but there are group differences at PND9; further, there are differences between Syn+-ir at PND5 vs. PND9. These differences would influence birth weight correlations.

Statistics (see above section as well):

- Table 2 indicates no significant differences between treatment groups, but it is stated: “The brain water fraction did not differ between treatment groups but was reduced in female pigs at PND9 (p = 0.03)”. Reduced compared to which group(s)? PND9 CON males? The statistical comparison is unclear.

- Two separate IGF-1 treatment schemes are utilized-continuous intra-arterial infusion for 5 days, and subcutaneous injection 3x/day for 9 days-but the reasoning for the difference in duration of treatment time is not given. Because PND9 piglets are older than PND5 piglets, comparisons between these groups should take into account age AND IGF-1 delivery mode (arguably, mode and duration of IGF-1 delivery is extremely important in this study, more so than the difference in age). Comparisons between CON@PND5 piglets and CON@PND9 piglets should be included for all outcomes.

All data and results from statistical comparisons should be reported:

- Figure 2 only contains [area %], but total number of NeuN+ neurons is reported in the text to not differ within “the total cortical area” at both time points and to not differ within the hippocampus at PND5, but no numbers or p-values are reported, and these data are not represented in Figure 2. The authors do not explain why %area differs between groups in the cortex, but number of NeuN+ neurons does not. Further, it is stated that both ventral and dorsal hippocampus were assessed, but data are then reported as simply “hippocampus”. Where data from ventral and dorsal combined and then compared? Only ROIs within the ventral hippocampus are represented in 2A.

- Figure 3&4: again Hippocampus is reported, but it is unclear if %area values across all sub-regions were summed to get this overall value. Unclear if ventral or dorsal or both.

- Extended data Figure 4-1A: text states Olig2+ cells (#) but graph indicates %. Data are missing for some ROIs (only 4 graphically represented, but the text indicates that at least 8 ROIs would have been assessed. Data for PND9 is missing.

- Fig 6: RPL23A expression should be confirmed with independent qPCR, as with CNMD and QRFPR. Data from PND9 should be presented in extended data tables or figures, as there is no reason to not show it.

- It is unclear whether IL6, IL10, and S100A9 were examined via independent qPCR or were extracted from the RNAseq dataset. Expression levels and stats should be reported.

- There is no logic to the data the authors decide to not show (indicated as “data not shown” in the text), data that are reported but not included, and data that are reported in extended data or directly in the text. All data should be included in some form (including statistical outcomes) if it is mentioned in the text.

- Images of IHC for only IGF1R, NeuN, Olig2, MBP, and DCX are shown; it would improve the manuscript if representative images of synaptophysin and Iba1 staining were also included, as these were quantified and compared between groups.

Minor revisions:

“necrotizing enterocolitis” should be removed as a keyword, as NEC was not a primary outcome of this study.

Significance statement, 1st sentence: “Deficits in systemic [IGF-1] are associated with...” instead of “is”.

Extended Data Table 1-1 (open field test) should be Extended Data Figure 1. Extended Data Figure 1-1 (antibodies) should be Extended Data Table 1. Extended Data Fig. 6-1 (primers) should be Extended Data Table 2. Extended Data Fig. 6-2 (DEGs in separate Excel file) should be Extended Data Table 3. Extended Data Fig. 1-2 (IGF1R immunofluorescence) should be Extended Data Fig. 2. Extended Data Fig. 4-1 (Olig2 IHC and IGF-1 signaling genes) should be Extended Data Fig 3. Reference to these extended data throughout the manuscript should be modified accordingly.

Extended Data Table 1-1B (which should be Extended Data Fig. 1C): figure legend needs to indicate that two representative heat maps are shown per group (CON vs. IGF-1), as there are four total heat maps.

Use of the term “neurological” score or analysis is confusing, as it indicates neural activity was measured. These should be replaced by “neuromotor” score or analysis.

Table 2 indicates no significant differences between treatment groups, but it is stated: “The brain water fraction did not differ between treatment groups but was reduced in female pigs at PND9 (p = 0.03)”. Reduced compared to which group(s)? PND9 CON males? The statistical comparison is unclear.

NEC lesion scoring system needs to be reported. Was fresh intestinal tissue scored immediately following euthanasia (or from photographs), or was tissue fixed then scored histologically?

Figure 3B-C: the green regression line for PND5 is difficult to see - suggest changing color.

Figure 6: In B, adjusted p-values should be presented in the graphs. In C, there is no explanation for why Opalin, AQP4, TTR, and EEF1A2 were selected for qPCR analysis from the entire RNAseq dataset.

Graphical representation of plasma IGF-1 levels in CON and supplemented piglets at PND5 and PND9 would be compelling; these values currently get lost in the text. Likewise, graphical representation of the correlation between protein synthesis rates and plasma IGF-1 levels would be nice to see.

Statistics

It is not clear why the authors opted to utilize multiple linear regression instead of two-way ANOVA/ANCOVA (time x treatment). Model design is confusing; it appears there were four groups: CON vs. IGF1 and PND5 vs. PND9 (as PND5 pigs received continuous i.a. treatment and PND9 pigs received s.c. treatment 3x/day). Interpretation of comparisons between groups is extremely confusing throughout the manuscript. At the very least, the authors need to justify why they chose to utilize multiple linear regression to analyze their datasets, and why treatment is included as a fixed effect (should be main effect), and litter, birth weight, and sex are included as fixed effects (instead of covariates?). Graphical representations of group comparisons are missing in some parts (for example, IGF1R expression across brain regions, especially as the authors claim differential expression), leading to further confusion. While sex is included in the model (as a fixed effect), it is unclear if sex differences were directly compared. While there are some regression analyses [correlations] graphically represented (ex: Fig3), the rest of the figures demonstrate group comparisons (CON vs IGF1 or PND7 vs PND9) and it is unclear what tests were used to acquire these p-values. The authors state that gut pathology was included as an interaction term for IHC analyses, but interactions between variables are not reported (whether significant or not).

## References

[B1] Afroz S, Parato J, Shen H, Smith SS (2016) Synaptic pruning in the female hippocampus is triggered at puberty by extrasynaptic GABAA receptors on dendritic spines. Elife 5:e15106. 10.7554/eLife.15106 27136678 PMC4871702

[B2] Anders S, Pyl PT, Huber W (2015) HTSeq–a Python framework to work with high-throughput sequencing data. Bioinformatics 31:166–169. 10.1093/bioinformatics/btu638 25260700 PMC4287950

[B3] Andersen AD, Sangild PT, Munch SL, van der Beek EM, Renes IB, Ginneken C, Greisen GO, Thymann T (2016) Delayed growth, motor function and learning in preterm pigs during early postnatal life. Am J Physiol Regul Integr Comp Physiol 310:R481–R492. 10.1152/ajpregu.00349.2015 26764054

[B4] Anderson SA, Classey JD, Condé F, Lund JS, Lewis DA (1995) Synchronous development of pyramidal neuron dendritic spines and parvalbumin-immunoreactive chandelier neuron axon terminals in layer III of monkey prefrontal cortex. Neuroscience 67:7–22. 10.1016/0306-4522(95)00051-j 7477911

[B5] Back SA (2017) White matter injury in the preterm infant: pathology and mechanisms. Acta Neuropathol 134:331–349. 10.1007/s00401-017-1718-6 28534077 PMC5973818

[B6] Back SA, Han BH, Luo NL, Chricton CA, Xanthoudakis S, Tam J, Arvin KL, Holtzman DM (2002) Selective vulnerability of late oligodendrocyte progenitors to hypoxia-ischemia. J Neurosci 22:455–463. 10.1523/JNEUROSCI.22-02-00455.2002 11784790 PMC6758669

[B7] Bæk O, Cilieborg MS, Nguyen DN, Bering SB, Thymann T, Sangild PT (2021) Sex-specific survival, growth, immunity and organ development in preterm pigs as models for immature newborns. Front Pediatr 9:626101. 10.3389/fped.2021.626101 33643975 PMC7905020

[B8] Bartlett WP, Li XS, Williams M (1992) Expression of IGF-1 mRNA in the murine subventricular zone during postnatal development. Brain Res Mol Brain Res 12:285–291. 10.1016/0169-328x(92)90131-t 1315903

[B9] Bauernfeind AL, Babbitt CC (2017) The predictive nature of transcript expression levels on protein expression in adult human brain. BMC Genomics 18:322. 10.1186/s12864-017-3674-x 28438116 PMC5402646

[B10] Beardsall K, Vanhaesebrouck S, Frystyk J, Ogilvy-Stuart AL, Vanhole C, van Weissenbruch M, Midgley P, Thio M, Cornette L, Gill B, Ossuetta I, Iglesias I, Theyskens C, de Jong M, Ahluwalia JS, de Zegher F, Dunger DB (2014) Relationship between insulin-like growth factor I levels, early insulin treatment, and clinical outcomes of very low birth weight infants. J Pediatr 164:1038–1044.e1. 10.1016/j.jpeds.2013.12.046 24518169

[B11] Beck KD, Powell-Braxton L, Widmer HR, Valverde J, Hefti F (1995) Igf1 gene disruption results in reduced brain size, CNS hypomyelination, and loss of hippocampal granule and striatal parvalbumin-containing neurons. Neuron 14:717–730. 10.1016/0896-6273(95)90216-3 7718235

[B12] Bellmund JL, Deuker L, Doeller CF (2019) Mapping sequence structure in the human lateral entorhinal cortex. Elife 8:e45333. 10.7554/eLife.45333 31383256 PMC6684227

[B13] Bourgeois JP, Jastreboff PJ, Rakic P (1989) Synaptogenesis in visual cortex of normal and preterm monkeys: evidence for intrinsic regulation of synaptic overproduction. Proc Natl Acad Sci U S A 86:4297–4301. 10.1073/pnas.86.11.4297 2726773 PMC287439

[B14] Bunn RC, King WD, Winkler MK, Fowlkes JL (2005) Early developmental changes in IGF-I, IGF-II, IGF binding protein-1, and IGF binding protein-3 concentration in the cerebrospinal fluid of children. Pediatr Res 58:89–93. 10.1203/01.PDR.0000156369.62787.96 15774848

[B15] Carro E, Spuch C, Trejo JL, Antequera D, Torres-Aleman I (2005) Choroid plexus megalin is involved in neuroprotection by serum insulin-like growth factor I. J Neurosci 25:10884–10893. 10.1523/JNEUROSCI.2909-05.2005 16306401 PMC6725866

[B16] Cheng CM, Reinhardt RR, Lee WH, Joncas G, Patel SC, Bondy CA (2000) Insulin-like growth factor 1 regulates developing brain glucose metabolism. Proc Natl Acad Sci U S A 97:10236–10241. 10.1073/pnas.170008497 10954733 PMC27834

[B17] Chrysis D, Calikoglu AS, Ye P, D'Ercole AJ (2001) Insulin-like growth factor-I overexpression attenuates cerebellar apoptosis by altering the expression of Bcl family proteins in a developmentally specific manner. J Neurosci 21:1481–1489. 10.1523/JNEUROSCI.21-05-01481.2001 11222638 PMC6762946

[B18] Clemmons DR (2016) Role of IGF binding proteins in regulating metabolism. Trends Endocrinol Metab 27:375–391. 10.1016/j.tem.2016.03.019 27117513

[B19] Czikk MJ, Totten S, Hammond R, Richardson BS (2015) Microtubule-associated protein 2 and synaptophysin in the preterm and near-term ovine fetal brain and the effect of intermittent umbilical cord occlusion. Reprod Sci 22:367–376. 10.1177/1933719114529371 24700051 PMC4352139

[B20] Davis TA, Fiorotto ML, Burrin DG, Vann RC, Reeds PJ, Nguyen HV, Beckett PR, Bush JA (2002) Acute IGF-I infusion stimulates protein synthesis in skeletal muscle and other tissues of neonatal pigs. Am J Physiol Endocrinol Metab 283:E638–E647. 10.1152/ajpendo.00081.2002 12217880

[B21] Deng JB, Yu DM, Li MS (2006) Formation of the entorhino-hippocampal pathway: a tracing study in vitro and in vivo. Neurosci Bull 22:305–314. 17690715

[B22] Dobbing J, Sands J (1979) Comparative aspects of the brain growth spurt. Early Hum Dev 3:79–83. 10.1016/0378-3782(79)90022-7 118862

[B23] Donato F, Jacobsen RI, Moser MB, Moser EI (2017) Stellate cells drive maturation of the entorhinal-hippocampal circuit. Science 355:eaai8178. 10.1126/science.aai8178 28154241

[B24] Duan W, Zhang YP, Hou Z, Huang C, Zhu H, Zhang CQ, Yin Q (2016) Novel insights into NeuN: from neuronal marker to splicing regulator. Mol Neurobiol 53:1637–1647. 10.1007/s12035-015-9122-5 25680637

[B25] Félix B, Léger ME, Albe-Fessard D, Marcilloux JC, Rampin O, Laplace JP (1999) Stereotaxic atlas of the pig brain. Brain Res Bull 49:1–137. 10.1016/s0361-9230(99)00012-x 10466025

[B26] Garcia-Segura LM, Rodriguez JR, Torres-Aleman I (1997) Localization of the insulin-like growth factor I receptor in the cerebellum and hypothalamus of adult rats: an electron microscopic study. J Neurocytol 26:479–490. 10.1023/a:1018581407804 9306246

[B27] Glantz LA, Gilmore JH, Hamer RM, Lieberman JA, Jarskog LF (2007) Synaptophysin and postsynaptic density protein 95 in the human prefrontal cortex from mid-gestation into early adulthood. Neuroscience 149:582–591. 10.1016/j.neuroscience.2007.06.036 17916412 PMC2128709

[B28] Gram M, Ekström C, Holmqvist B, Carey G, Wang X, Vallius S, Hellström W, Ortenlöf N, Agyemang AA, Smith LEH, Hellström A, Mangili A, Barton N, Ley D (2021) Insulin-like growth factor 1 in the preterm rabbit pup: characterization of cerebrovascular maturation following administration of recombinant human insulin-like growth factor 1/insulin-like growth factor 1-binding protein 3. Dev Neurosci 43:281–295. 10.1159/000516665 34218224 PMC8623584

[B29] Hansen-Pupp I, Hellström-Westas L, Cilio CM, Andersson S, Fellman V, Ley D (2007) Inflammation at birth and the insulin-like growth factor system in very preterm infants. Acta Paediatr 96:830–836. 10.1111/j.1651-2227.2007.00276.x 17465986

[B30] Hansen-Pupp I, Hövel H, Hellström A, Hellström-Westas L, Löfqvist C, Larsson EM, Lazeyras F, Fellman V, Hüppi PS, Ley D (2011) Postnatal decrease in circulating insulin-like growth factor-I and low brain volumes in very preterm infants. J Clin Endocrinol Metab 96:1129–1135. 10.1210/jc.2010-2440 21289247

[B31] Hansen-Pupp I, Hövel H, Löfqvist C, Hellström-Westas L, Fellman V, Hüppi PS, Hellström A, Ley D (2013) Circulatory insulin-like growth factor-I and brain volumes in relation to neurodevelopmental outcome in very preterm infants. Pediatr Res 74:564–569. 10.1038/pr.2013.135 23942554

[B32] Hellström A, Engström E, Hård AL, Albertsson-Wikland K, Carlsson B, Niklasson A, Löfqvist C, Svensson E, Holm S, Ewald U, Holmström G, Smith LE (2003) Postnatal serum insulin-like growth factor I deficiency is associated with retinopathy of prematurity and other complications of premature birth. Pediatrics 112:1016–1020. 10.1542/peds.112.5.1016 14595040

[B33] Hellström A, Ley D, Hansen-Pupp I, Hallberg B, Löfqvist C, van Marter L, van Weissenbruch M, Ramenghi LA, Beardsall K, Dunger D, Hård AL, Smith LE (2016) Insulin-like growth factor 1 has multisystem effects on foetal and preterm infant development. Acta Paediatr 105:576–586. 10.1111/apa.13350 26833743 PMC5069563

[B34] Holgersen K, Gao X, Narayanan R, Gaur T, Carey G, Barton N, Pan X, Muk T, Thymann T, Sangild PT (2020) Supplemental insulin-like growth factor-1 and necrotizing enterocolitis in preterm pigs. Front Pediatr 8:602047. 10.3389/fped.2020.602047 33614541 PMC7891102

[B35] Holgersen K, Rasmussen MB, Carey G, Burrin DG, Thymann T, Sangild PT (2022) Clinical outcome and gut development after insulin-like growth factor-1 supplementation to preterm pigs. Front Pediatr 10:868911. 10.3389/fped.2022.868911 35989990 PMC9389362

[B36] Holme Nielsen C, Bladt Brandt A, Thymann T, Obelitz-Ryom K, Jiang P, Vanden Hole C, van Ginneken C, Pankratova S, Sangild PT (2018) Rapid postnatal adaptation of neurodevelopment in pigs born late preterm. Dev Neurosci 40:586–600. 10.1159/000499127 31141813

[B37] Horsch S, Parodi A, Hallberg B, Malova M, Bjorkman-Burtscher IM, Hansen-Pupp I, Marlow N, Beardsall K, Dunger D, van Weissenbruch M, Smith LEH, Hamdani M, Mangili A, Barton N, Ramenghi LA, Hellstrom A, Ley D (2020) Randomized control trial of postnatal rhIGF-1/rhIGFBP-3 replacement in preterm infants: *post-hoc* analysis of its effect on brain injury. Front Pediatr 8:517207. 10.3389/fped.2020.517207 33163463 PMC7581737

[B38] Ishibashi N, Scafidi J, Murata A, Korotcova L, Zurakowski D, Gallo V, Jonas RA (2012) White matter protection in congenital heart surgery. Circulation 125:859–871. 10.1161/CIRCULATIONAHA.111.048215 22247493 PMC3288390

[B39] Jin J, Ravindran P, Di Meo D, Püschel AW (2019) Igf1R/InsR function is required for axon extension and corpus callosum formation. PLoS One 14:e0219362. 10.1371/journal.pone.0219362 31318893 PMC6638864

[B40] Kajantie E, Dunkel L, Rutanen EM, Seppälä M, Koistinen R, Sarnesto A, Andersson S (2002) IGF-I, IGF binding protein (IGFBP)-3, phosphoisoforms of IGFBP-1, and postnatal growth in very low birth weight infants. J Clin Endocrinol Metab 87:2171–2179. 10.1210/jcem.87.5.8457 11994360

[B41] Kappeler L, De Magalhaes Filho C, Dupont J, Leneuve P, Cervera P, Périn L, Loudes C, Blaise A, Klein R, Epelbaum J, Le Bouc Y, Holzenberger M (2008) Brain IGF-1 receptors control mammalian growth and lifespan through a neuroendocrine mechanism. PLoS Biol 6:e254. 10.1371/journal.pbio.0060254 18959478 PMC2573928

[B42] Kim D, Langmead B, Salzberg SL (2015) HISAT: a fast spliced aligner with low memory requirements. Nat Methods 12:357–360. 10.1038/nmeth.3317 25751142 PMC4655817

[B43] Labandeira-Garcia JL, Costa-Besada MA, Labandeira CM, Villar-Cheda B, Rodriguez-Perez AI (2017) Insulin-like growth factor-1 and neuroinflammation. Front Aging Neurosci 9:365. 10.3389/fnagi.2017.00365 29163145 PMC5675852

[B44] Langford K, Nicolaides K, Miell JP (1998) Maternal and fetal insulin-like growth factors and their binding proteins in the second and third trimesters of human pregnancy. Hum Reprod 13:1389–1393. 10.1093/humrep/13.5.1389 9647578

[B45] Lavezzi AM, Corna MF, Matturri L (2013) Neuronal nuclear antigen (NeuN): a useful marker of neuronal immaturity in sudden unexplained perinatal death. J Neurol Sci 329:45–50. 10.1016/j.jns.2013.03.012 23570982

[B46] Liegl R, Löfqvist C, Hellström A, Smith LE (2016) IGF-1 in retinopathy of prematurity, a CNS neurovascular disease. Early Hum Dev 102:13–19. 10.1016/j.earlhumdev.2016.09.008 27650433 PMC5085844

[B47] Lineham JD, Smith RM, Dahlenburg GW, King RA, Haslam RR, Stuart MC, Faull L (1986) Circulating insulin-like growth factor I levels in newborn premature and full-term infants followed longitudinally. Early Hum Dev 13:37–46. 10.1016/0378-3782(86)90096-4 3956421

[B48] Liu W, Ye P, O'Kusky JR, D'Ercole AJ (2009) Type 1 insulin-like growth factor receptor signaling is essential for the development of the hippocampal formation and dentate gyrus. J Neurosci Res 87:2821–2832. 10.1002/jnr.22129 19437543

[B49] Liu Y, Bergmann T, Mori Y, Peralvo Vidal JM, Pihl M, Vasistha NA, Thomsen PD, Seemann SE, Gorodkin J, Hyttel P, Khodosevich K, Witter MP, Hall VJ (2021) Development of the entorhinal cortex occurs via parallel lamination during neurogenesis. Front Neuroanat 15:663667. 10.3389/fnana.2021.663667 34025365 PMC8139189

[B50] Löfqvist C, Hellgren G, Niklasson A, Engström E, Ley D, Hansen-Pupp I (2012) Low postnatal serum IGF-I levels are associated with bronchopulmonary dysplasia (BPD). Acta Paediatr 101:1211–1216. 10.1111/j.1651-2227.2012.02826.x 22924869 PMC3569611

[B51] Love MI, Huber W, Anders S (2014) Moderated estimation of fold change and dispersion for RNA-seq data with DESeq2. Genome Biol 15:550. 10.1186/s13059-014-0550-8 25516281 PMC4302049

[B52] Marks JL, Porte D Jr, Baskin DG (1991) Localization of type I insulin-like growth factor receptor messenger RNA in the adult rat brain by in situ hybridization. Mol Endocrinol 5:1158–1168. 10.1210/mend-5-8-1158 1658638

[B53] Masliah E, Terry RD, Alford M, DeTeresa R (1990) Quantitative immunohistochemistry of synaptophysin in human neocortex: an alternative method to estimate density of presynaptic terminals in paraffin sections. J Histochem Cytochem 38:837–844. 10.1177/38.6.2110586 2110586

[B54] Mullen RJ, Buck CR, Smith AM (1992) NeuN, a neuronal specific nuclear protein in vertebrates. Development 116:201–211. 10.1242/dev.116.1.201 1483388

[B55] Nguyen DN, Jiang P, Frøkiær H, Heegaard PM, Thymann T, Sangild PT (2016) Delayed development of systemic immunity in preterm pigs as a model for preterm infants. Sci Rep 6:36816. 10.1038/srep36816 27830761 PMC5103294

[B56] Nieto-Estévez V, Oueslati-Morales CO, Li L, Pickel J, Morales AV, Vicario-Abejón C (2016) Brain insulin-like growth factor-I directs the transition from stem cells to mature neurons during postnatal/adult hippocampal neurogenesis. Stem Cells 34:2194–2209. 10.1002/stem.2397 27144663

[B57] Nishijima T, Piriz J, Duflot S, Fernandez AM, Gaitan G, Gomez-Pinedo U, Verdugo JM, Leroy F, Soya H, Nuñez A, Torres-Aleman I (2010) Neuronal activity drives localized blood-brain-barrier transport of serum insulin-like growth factor-I into the CNS. Neuron 67:834–846. 10.1016/j.neuron.2010.08.007 20826314

[B58] Pan J, Ruest LB, Xu S, Wang E (2004) Immuno-characterization of the switch of peptide elongation factors eEF1A-1/EF-1alpha and eEF1A-2/S1 in the central nervous system during mouse development. Brain Res Dev Brain Res 149:1–8. 10.1016/j.devbrainres.2003.10.011 15013623 PMC2830753

[B59] Pan Z, Zhang J, Zhang J, Zhou B, Chen J, Jiang Z, Liu H (2012) Expression profiles of the insulin-like growth factor system components in liver tissue during embryonic and postnatal growth of Erhualian and Yorkshire reciprocal cross F1 pigs. Asian-Australas J Anim Sci 25:903–912. 10.5713/ajas.2011.11385 25049643 PMC4092980

[B60] Paolicelli RC, Bolasco G, Pagani F, Maggi L, Scianni M, Panzanelli P, Giustetto M, Ferreira TA, Guiducci E, Dumas L, Ragozzino D, Gross CT (2011) Synaptic pruning by microglia is necessary for normal brain development. Science 333:1456–1458. 10.1126/science.1202529 21778362

[B61] Petanjek Z, Judaš M, Šimic G, Rasin MR, Uylings HB, Rakic P, Kostovic I (2011) Extraordinary neoteny of synaptic spines in the human prefrontal cortex. Proc Natl Acad Sci U S A 108:13281–13286. 10.1073/pnas.1105108108 21788513 PMC3156171

[B62] Popken GJ, Hodge RD, Ye P, Zhang J, Ng W, O’Kusky JR, D'Ercole AJ (2004) In vivo effects of insulin-like growth factor-I (IGF-I) on prenatal and early postnatal development of the central nervous system. Eur J Neurosci 19:2056–2068. 10.1111/j.0953-816X.2004.03320.x 15090033

[B63] Ronaghi A, Zibaii MI, Pandamooz S, Nourzei N, Motamedi F, Ahmadiani A, Dargahi L (2019) Entorhinal cortex stimulation induces dentate gyrus neurogenesis through insulin receptor signaling. Brain Res Bull 144:75–84. 10.1016/j.brainresbull.2018.11.011 30472148

[B64] Rudar M, Naberhuis JK, Suryawan A, Nguyen HV, Stoll B, Style CC, Verla MA, Olutoye OO, Burrin DG, Fiorotto ML, Davis TA (2021) Intermittent bolus feeding does not enhance protein synthesis, myonuclear accretion, or lean growth more than continuous feeding in a premature piglet model. Am J Physiol Endocrinol Metab 321:E737–E752. 10.1152/ajpendo.00236.2021 34719946 PMC8714968

[B65] Saikali S, Meurice P, Sauleau P, Eliat PA, Bellaud P, Randuineau G, Vérin M, Malbert CH (2010) A three-dimensional digital segmented and deformable brain atlas of the domestic pig. J Neurosci Methods 192:102–109. 10.1016/j.jneumeth.2010.07.041 20692291

[B66] Sangild PT, Thymann T, Schmidt M, Stoll B, Burrin DG, Buddington RK (2013) Invited review: the preterm pig as a model in pediatric gastroenterology. J Anim Sci 91:4713–4729. 10.2527/jas.2013-6359 23942716 PMC3984402

[B67] Schwanhäusser B, Busse D, Li N, Dittmar G, Schuchhardt J, Wolf J, Chen W, Selbach M (2011) Global quantification of mammalian gene expression control. Nature 473:337–342. 10.1038/nature10098 21593866

[B68] Skranes J, Løhaugen GC, Evensen KA, Indredavik MS, Haraldseth O, Dale AM, Brubakk AM, Martinussen M (2012) Entorhinal cortical thinning affects perceptual and cognitive functions in adolescents born preterm with very low birth weight (VLBW). Early Hum Dev 88:103–109. 10.1016/j.earlhumdev.2011.07.017 21839590

[B69] Soleimani F, Zaheri F, Abdi F (2014) Long-term neurodevelopmental outcomes after preterm birth. Iran Red Crescent Med J 16:e17965. 10.5812/ircmj.17965 25068052 PMC4102985

[B70] Stinnett GR, Lin S, Korotcov AV, Korotcova L, Morton PD, Ramachandra SD, Pham A, Kumar S, Agematsu K, Zurakowski D, Wang PC, Jonas RA, Ishibashi N (2017) Microstructural alterations and oligodendrocyte dysmaturation in white matter after cardiopulmonary bypass in a juvenile porcine model. J Am Heart Assoc 6:e005997. 28862938 10.1161/JAHA.117.005997 PMC5586442

[B71] Suh HS, Zhao ML, Derico L, Choi N, Lee SC (2013) Insulin-like growth factor 1 and 2 (IGF1, IGF2) expression in human microglia: differential regulation by inflammatory mediators. J Neuroinflammation 10:37. 10.1186/1742-2094-10-37 23497056 PMC3607995

[B72] Sun J, Pan X, Christiansen LI, Yuan XL, Skovgaard K, Chatterton DEW, Kaalund SS, Gao F, Sangild PT, Pankratova S (2018) Necrotizing enterocolitis is associated with acute brain responses in preterm pigs. J Neuroinflammation 15:180. 10.1186/s12974-018-1201-x 29885660 PMC5994241

[B73] Unal-Cevik I, Kilinç M, Gürsoy-Ozdemir Y, Gurer G, Dalkara T (2004) Loss of NeuN immunoreactivity after cerebral ischemia does not indicate neuronal cell loss: a cautionary note. Brain Res 1015:169–174. 10.1016/j.brainres.2004.04.032 15223381

[B74] Valeeva G, Janackova S, Nasretdinov A, Rychkova V, Makarov R, Holmes GL, Khazipov R, Lenck-Santini PP (2019) Emergence of coordinated activity in the developing entorhinal-hippocampal network. Cereb Cortex 29:906–920. 10.1093/cercor/bhy309 30535003 PMC6319314

[B75] Vieira M, Gomes JR, Saraiva MJ (2015) Transthyretin induces insulin-like growth factor I nuclear translocation regulating its levels in the hippocampus. Mol Neurobiol 51:1468–1479. 10.1007/s12035-014-8824-4 25084758 PMC4434863

[B76] Vieira M, Leal SS, Gomes CM, Saraiva MJ (2016) Evidence for synergistic action of transthyretin and IGF-I over the IGF-I receptor. Biochim Biophys Acta 1862:797–804. 10.1016/j.bbadis.2016.01.008 26804653

[B77] Ye P, Carson J, D'Ercole AJ (1995) *In vivo* actions of insulin-like growth factor-I (IGF-I) on brain myelination: studies of IGF-I and IGF binding protein-1 (IGFBP-1) transgenic mice. J Neurosci 15:7344–7356. 10.1523/JNEUROSCI.15-11-07344.1995 7472488 PMC6578047

[B78] Ye P, Li L, Richards RG, DiAugustine RP, D'Ercole AJ (2002) Myelination is altered in insulin-like growth factor-I null mutant mice. J Neurosci 22:6041–6051. 10.1523/JNEUROSCI.22-14-06041.2002 12122065 PMC6757955

[B79] Zeger M, Popken G, Zhang J, Xuan S, Lu QR, Schwab MH, Nave KA, Rowitch D, D'Ercole AJ, Ye P (2007) Insulin-like growth factor type 1 receptor signaling in the cells of oligodendrocyte lineage is required for normal in vivo oligodendrocyte development and myelination. Glia 55:400–411. 10.1002/glia.20469 17186502 PMC1774584

[B80] Zehr JL, Todd BJ, Schulz KM, McCarthy MM, Sisk CL (2006) Dendritic pruning of the medial amygdala during pubertal development of the male Syrian hamster. J Neurobiol 66:578–590. 10.1002/neu.20251 16555234

[B81] Zhang K, Sejnowski TJ (2000) A universal scaling law between gray matter and white matter of cerebral cortex. Proc Natl Acad Sci U S A 97:5621–5626. 10.1073/pnas.090504197 10792049 PMC25878

[B82] Ziegler AN, Levison SW, Wood TL (2015) Insulin and IGF receptor signalling in neural-stem-cell homeostasis. Nat Rev Endocrinol 11:161–170. 10.1038/nrendo.2014.208 25445849 PMC5513669

