# Supplementary material for: Insulin-Like Growth Factor-1 Supplementation Promotes Brain Maturation in Preterm Pigs
Source: eNeuro. 2023 Apr 13;10(4):ENEURO.0430-22.2023. doi: 10.1523/ENEURO.0430-22.2023 (PMC10112548; doi:10.1523/ENEURO.0430-22.2023)
Supplement: Figure 1-1 — List of primary and secondary antibodies, dilutions, and supplier information. Download Figure 1-1, DOCX file. [file enu-eN-NWR-0430-22-s07.docx]

**Extended Data Fig. 1-1.** List of primary and secondary antibodies, dilutions, and supplier information.

| Antibody name | Source | Dilution | Supplier (#Catalog) |
| --- | --- | --- | --- |
| *Primary antibody* | | | |
| DCX | Rabbit polyclonal | 0.4 µg/ml | Abcam (#ab18723) |
| IBA1 | Goat polyclonal | 1:750 | Abcam (#ab5076) |
| IGF1R  IGF1R | Goat polyclonal or  Mouse monoclonal | 8 µg/ml  1:400 | R&D systems (#AF-305-NA)/ Thermo Fisher (#3G5C1) |
| GFAP | Chicken polyclonal | 1:4000 | Abcam (ab4674) |
| MBP | Mouse monoclonal | 1:50 | BioLegend (#836504) |
| NeuN | Mouse monoclonal | 1:150 | Merck (#MAB377) |
| Olig2 | Rabbit polyclonal | 1:2000 | Merck (#AB9610) |
| Synaptophysin | Mouse monoclonal | 1:100 | Abcam (#ab8049) |
| *Secondary antibody* |  |  |  |
| Rabbit IgG/HRP | Goat | 1:1 | Vector (#MP7451) |
| Mouse IgG/HRP | Goat | 1:1 | Vector (#MP7452) |
| Rabbit IgG/AF | Donkey | 1:200 | Jackson IR (711-546-152, 711-296-152 or 711-606-152) |
| Mouse IgG/AF | Donkey | 1:200 | Jackson IR (715-546-150, 715-296-150 or 715-606-150) |
| Chicken IgG/AF | Donkey | 1:200 | Jackson IR (703-546-155) |
