# Supplementary figures and images for: Insulin-Like Growth Factor-1 Supplementation Promotes Brain Maturation in Preterm Pigs
Source: eNeuro. 2023 Apr 13;10(4):ENEURO.0430-22.2023. doi: 10.1523/ENEURO.0430-22.2023 (PMC10112548; doi:10.1523/ENEURO.0430-22.2023)

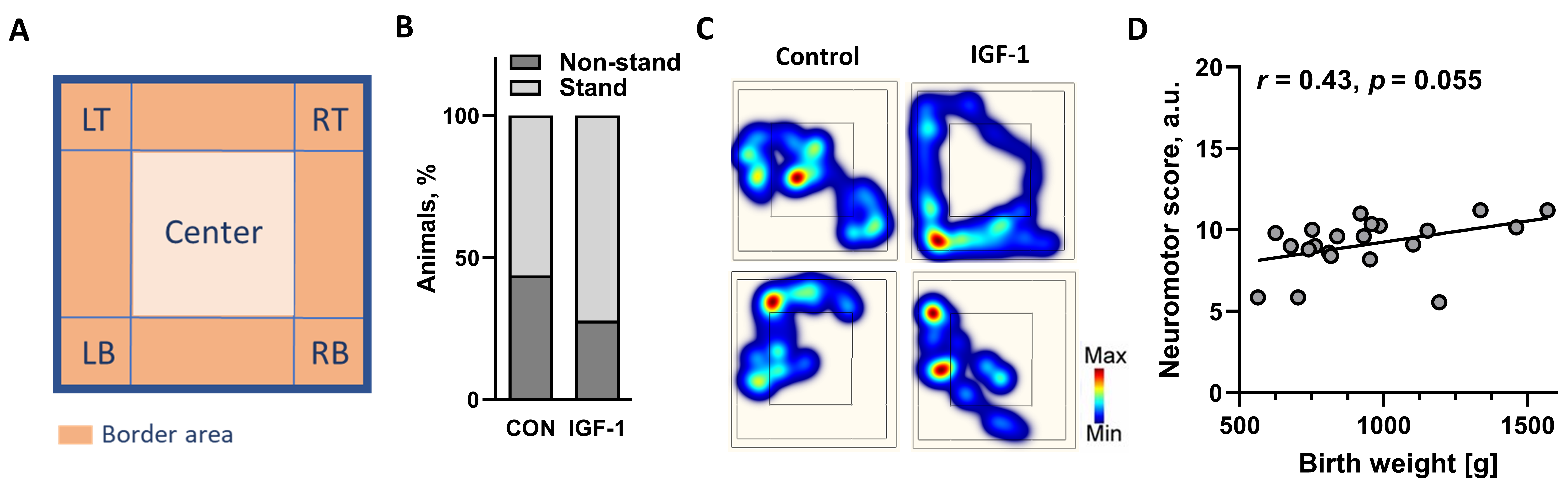

Supplement: Table 1-1 — Open-field test at postnatal day 7. A, Location and annotation of virtual zones in the open field arena, LT, RT, RB, and LB. B, The number of animals able to stand at postnatal day 7 was not different between the CON (n = 16) and IGF-1 groups (n = 18). C, To illustrate the cumulative tracks of pigs in the arena over 4 min for the IGF-1 and control groups, two representative open field heatmaps are shown for each group. Blue to red indicates low to high occupancy. D, Correlation between birth weight and neuromotor score at postnatal day 7. Correlation data were analyzed by Pearson R correlation analysis, p < 0.05 is considered statistically significant. Download Table 1-1, TIF file. [file enu-eN-NWR-0430-22-s05.tif]

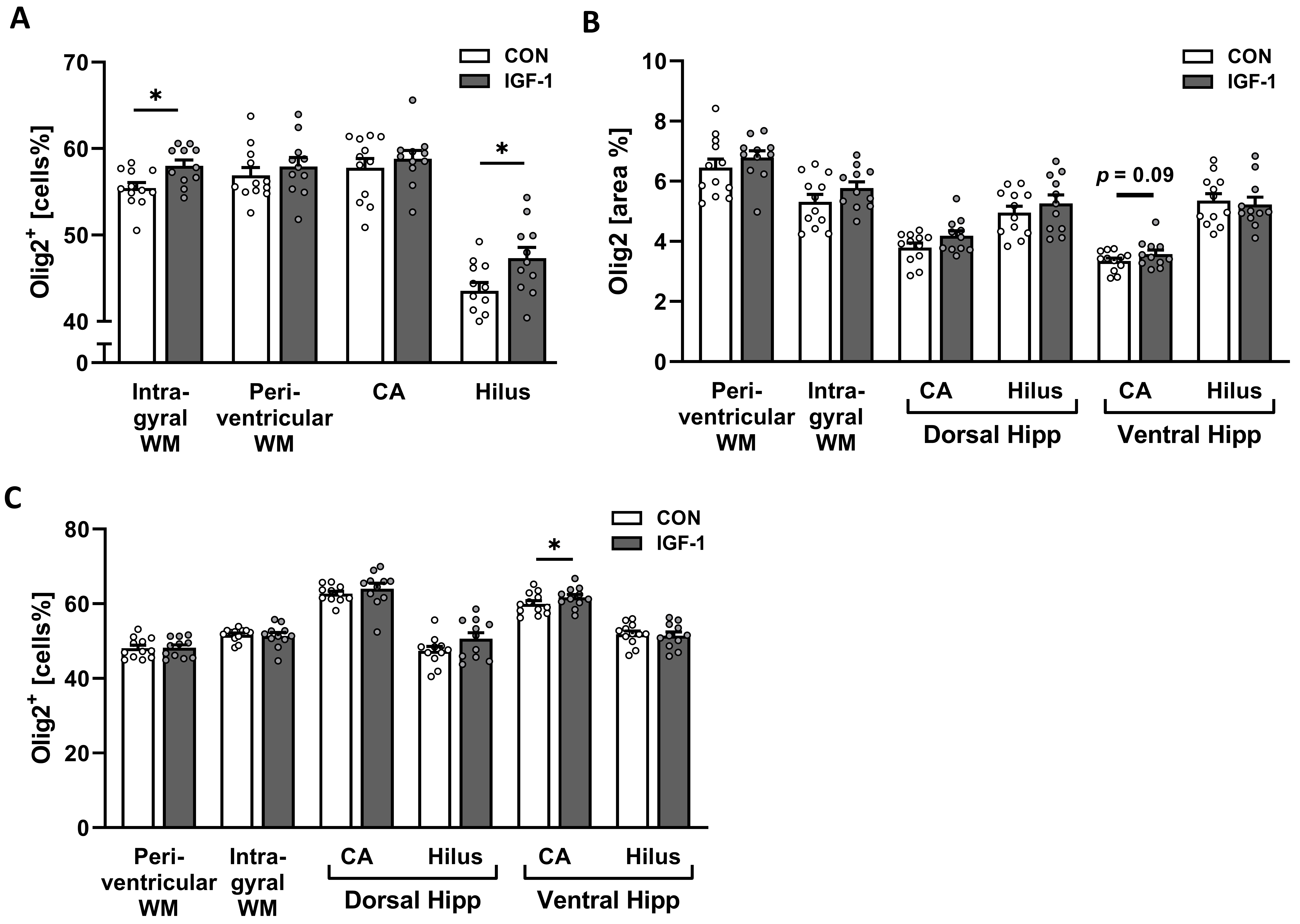

Supplement: Figure 4-1 — A, Quantification of the number of Olig2+ cells in the IGWM (average of temporal, cingulate, and parietal subcortical tracts), PvWM, CA, and hilar region (both average of respective dorsal and ventral hippocampal quantifications) of the hippocampus at postnatal day 5. CON, n = 12; IGF-1, n = 11. B, C, Quantification of the area of Olig2+-IR (B) and the number of Olig2+ cells (C) on postnatal day 9 in the PvWM, IGWM (average of temporal, cingulate, and parietal subcortical tracts), dorsal and ventral CA, and hilus of the hippocampus (Hipp). All results are expressed as the mean ± SEM. All data were analyzed using a linear model for each time point; statistically significant effects of treatment are shown as *p < 0.05. Download Figure 4-1, TIF file. [file enu-eN-NWR-0430-22-s03.tif]

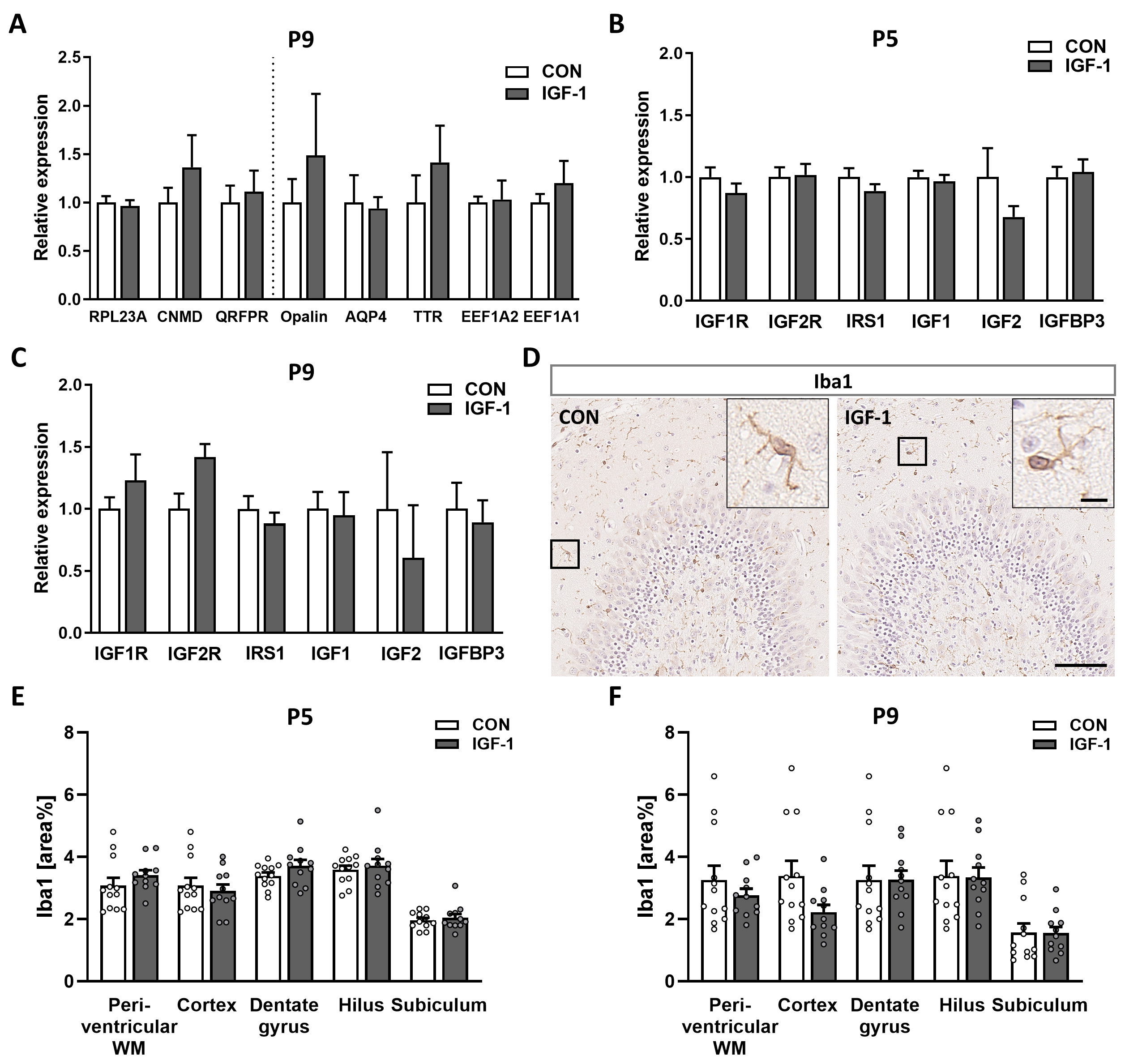

Supplement: Figure 6-3 — A, C, Relative expression (fold change compared with CON) of selected genes (A) and genes related to IGF-1 signaling in the hippocampus on P9 analyzed by qPCR (C). n = 7–8/treatment group. B, Relative expression (fold change compared to CON) of genes related to IGF-1 signaling in the hippocampus of P5 pigs analyzed by qPCR. n = 11–13/treatment group. D, Representative images of Iba1/hematoxylin-labeled dentate gyrus on P5 from pigs treated with vehicle (CON) or IGF-1. Scale bars: D, 100 μm; inset, 10 μm. E, F, Quantification of the area of Iba1-IR on P5 (E) and P9 (F) in the PvWM, cortex, dentate gyrus (dorsal + ventral), hilus (dorsal + ventral), and subiculum. All results are expressed as the mean ± SEM. All data were analyzed using a linear model for each time point. Download Figure 6-3, TIF file. [file enu-eN-NWR-0430-22-s04.tif]
