# Supplementary material for: Insulin-Like Growth Factor-1 Supplementation Promotes Brain Maturation in Preterm Pigs
Source: eNeuro. 2023 Apr 13;10(4):ENEURO.0430-22.2023. doi: 10.1523/ENEURO.0430-22.2023 (PMC10112548; doi:10.1523/ENEURO.0430-22.2023)
Supplement: Figure 6-1 — List of primers for qPCR. F, Forward primer; R, reverse primer; ¤, primers for these genes were adopted from Pan et al. (2012). Download Figure 6-1, DOCX file. [file enu-eN-NWR-0430-22-s08.docx]

**Extended Data Fig. 6-1.** List of primers for qPCR.

| **Gene symbol** | **Gene name** | **Primer sequences (5’ to 3’)** | **Product size (bp)** |
| --- | --- | --- | --- |
| AQP4 | Aquaporin 4 | **F:** GAGCCGGGATTCTCTACCTG  **R:** ATTTCCATGAACCGTGGTGA | 74 |
| CNMD | Chondromodulin | **F:** CATGACGTTCGACCCTCGAC  **R:** CGGCAGCCTTGGTAGTTGTA | 135 |
| EEF1A1 | Eukaryotic translation elongation factor 1 alpha 1 | **F:** ttgcattctaccaccaactcgt  **R:** aacattgactggagcaaaggtg | 157 |
| EEF1A2 | Eukaryotic translation elongation factor 1 alpha 2 | **F:** aacgtgtcagtcaaggacatcc  **R:** cgggtggttcagaatgatga | 189 |
| HPRT1 | Hypoxanthine phosphoribosyltransferase 1 | **F:** ACACTGGCAAAACAATGCAA  **R:** TGCAACCTTGACCATCTTTG | 71 |
| IGF1^¤^ | Insulin-like growth factor 1 | **F:** ATTTCTTGAAGGTAAAGATGCA  **R:** CAGCCCCACAGAGGGTCTCA | 117 |
| IGF2^¤^ | Insulin-like growth factor 2 | **F:** CCCAGTGAGACTCTGTGCG  **R:** CAGGTGTCATAGCGGAAGAAC | 275 |
| IGFBP-3^¤^ | insulin-like growth factor-binding protein 3 | **F:** GACACGCTGAACCACCTCA  **R:** CGTACTTATCCACGCACCAG | 151 |
| IGF1R^¤^ | Insulin-like growth factor 1 receptor | **F:** CGAGAGACATCTATGAGACA  **R:** TCCTCACTGTAGTAGAAGGA | 382 |
| IGF2R^¤^ | Insulin-like growth factor 2 receptor | **F:** ATCCTCAATCCCATAGCC  **R:** CTCTTACAATGAAACGCAAT | 110 |
| IL-6 | Interleukin 6 | **F:** TGGGTTCAATCAGGAGACCT  **R:** CAGCCTCGACATTTCCCTTA | 116 |
| IL-10 | Interleukin 10 | **F:** GTCCGACTCAACGAAGAAGG  **R:** GCCAGGAAGATCAGGCAATA | 73 |
| IRS1 | Insulin-receptor substrate 1 | **F:** TGGACATCACAGCAGAATGAAGA  **R:** GGTGTGAGGTCCTGGTTGTG | 105 |
| OPALIN | Myelin paranodal and inner loop protein | **F:** CTGCTGGTGGCCTTACTGTT  **R:** TTCACAAGGCCTCTCGATTT | 84 |
| S100A9 | S100 calcium-binding protein A9 | **F:** TGCCAATCAAGACGAACAGGT  **R:** ACTCCTTGTGGATGTTGTCGT | 92 |
| TTR | Transthyretin | **F:** TGCTGGTGAATCCAAGTGTC  **R:** CACTTTCACGCCTACGTTCA | 85 |
| QRFPR | Pyroglutamylated RFamide peptide receptor | **F:** GTCTGGTTGGTGGCAGTCAT  **R:** GATTTTCTGGTGCACAGGGC | 135 |

F, forward primer; R, reverse primer; ^¤^, primers for these genes were adopted from (Pan et al., 2012).
